# Lactate supply overtakes glucose when neural computational and cognitive loads scale up

**DOI:** 10.1073/pnas.2212004119

**Published:** 2022-11-14

**Authors:** Yulia Dembitskaya, Charlotte Piette, Sylvie Perez, Hugues Berry, Pierre J. Magistretti, Laurent Venance

**Affiliations:** ^a^Dynamics and Pathophysiology of Neuronal Networks Team, Center for Interdisciplinary Research in Biology (CIRB), Collège de France, CNRS, INSERM, Université PSL, 75005 Paris, France;; ^b^AIStroSight Lab, INRIA, Hospices Civils de Lyon, Université Claude Bernard Lyon 1, 69603 Villeurbanne, France;; ^c^University of Lyon, LIRIS UMR5205, 69622 Villeurbanne, France;; ^d^Biological and Environmental Sciences and Engineering (BESE) Division, King Abdullah University of Science and Technology (KAUST), 23955-6900 Thuwal, Saudi Arabia;; ^e^Brain Mind Institute, EPFL, 1015 Lausanne, Switzerland

**Keywords:** neuroenergetic, synaptic plasticity, learning and memory, lactate, glucose

## Abstract

Neural computational power is determined by neuroenergetics, but how and which energy substrates are allocated to various forms of memory engram is unclear. To solve this question, we asked whether neuronal fueling by glucose or lactate scales differently upon increasing neural computation and cognitive loads. Here, using electrophysiology, two-photon imaging, cognitive tasks, and mathematical modeling, we show that both glucose and lactate are involved in engram formation, with lactate supporting long-term synaptic plasticity evoked by high-stimulation load activity patterns and high attentional load in cognitive tasks and glucose being sufficient for less demanding neural computation and learning tasks. Indeed, we show that lactate is mandatory for demanding neural computation, such as theta-burst stimulation, while glucose is sufficient for lighter forms of activity-dependent long-term potentiation (LTP), such as spike timing–dependent plasticity (STDP). We find that subtle variations of spike number or frequency in STDP are sufficient to shift the on-demand fueling from glucose to lactate. Finally, we demonstrate that lactate is necessary for a cognitive task requiring high attentional load, such as the object-in-place task, and for the corresponding in vivo hippocampal LTP expression but is not needed for a less demanding task, such as a simple novel object recognition. Overall, these results demonstrate that glucose and lactate metabolism are differentially engaged in neuronal fueling depending on the complexity of the activity-dependent plasticity and behavior.

## Significance

Brain activity and performance are constrained by energy metabolism. Glucose and lactate have been proposed as energy substrates for neurons allocated to various forms of memory. We demonstrate that glucose and lactate metabolism are differentially engaged in neuronal fueling depending on the activity-dependent plasticity and behavioral complexity. These results reconcile a decades-long debate regarding the nature of the neuroenergetic sources used by synaptic activity with the potential of inspiring future lines of research regarding neuroenergetic rules. The brain has high energy demands, and alterations in neuroenergetics are hallmarks of several neuropathologies. A better knowledge of the cellular and molecular mechanisms of neuroenergetics, as reported here, may be instructive in targeting energy metabolism deficits as a therapeutic approach for neurodegenerative diseases.

Brain activity and performance are tightly constrained by neurovasculature–neuroenergetic coupling (1–3). Neuroenergetics, that is, brain energy metabolism, relies on the blood supply of glucose from the circulation. Evidence accrued over the last two decades has indicated that blood glucose is taken up during synaptic activity (4, 5), mainly by glial cells (astrocytes and oligodendrocytes), and metabolized by aerobic glycolysis, resulting in the release of lactate before transport to neurons as an energy substrate (6–13) necessary for optimized neuronal coding and memory consolidation (14–22). When astrocytes constitute the source of lactate, this process is known as the astrocyte–neuron lactate shuttle in which lactate is transferred from astrocytes to neurons through monocarboxylate transporters, providing an energy substrate for neurons (7). Indeed, lactate can be rapidly metabolized to pyruvate, enter the tricarboxylic acid cycle, and feed the mitochondrial respiratory chain to produce ATP. Other fates of glucose include its glial storage in the form glycogen (7, 23, 24); some degree of glucose uptake occurs in neurons via transporters mainly aimed at feeding the pentose phosphate shunt to produce reducing equivalents (25–27), which is involved in olfactory memory in *Drosophila* (28). Nevertheless, the nature of the energy substrate, glucose versus lactate, allocated to various forms of memory engram and cognitive load is not known.

Here, we tested various forms of activity patterns (rate- and time-coding) for Hebbian long-term synaptic plasticity expression in rat cornu ammonis 1 (CA1) hippocampal pyramidal cells and behavioral tasks with increasing cognitive loads to determine under which conditions glucose and/or lactate are crucial for engram formation and memory. To this end, using brain slice and in vivo electrophysiology, two-photon imaging, mathematical modeling, and recognition memory tasks, we show that astrocytic lactate is mandatory for demanding neural computation, while glucose is sufficient for lighter forms of activity-dependent long-term potentiation (LTP) and that subtle variations of action potential amount or frequency are sufficient to direct the energetic dependency from glucose to lactate. Furthermore, we demonstrate that lactate is necessary for a cognitive task requiring high attentional load (object-in-place [OiP] task) and for the corresponding in vivo hippocampal potentiation but is not needed for a less demanding task (novel object recognition [NOR]). Our results demonstrate that glucose and lactate metabolism are differentially engaged in neuronal fueling depending on the complexity of the activity-dependent plasticity and behavior. Beyond reconciling a decades-long debate (7, 11, 26, 27), our results demonstrate the importance of distinguishing specific cellular and molecular mechanisms because the corresponding cognitive perturbations might depend on whether lactate or glucose metabolism is perturbed.

## Results

### Rate and Time Coding Rely Differently on Lactate Availability to Neurons.

**Fig. 1.**
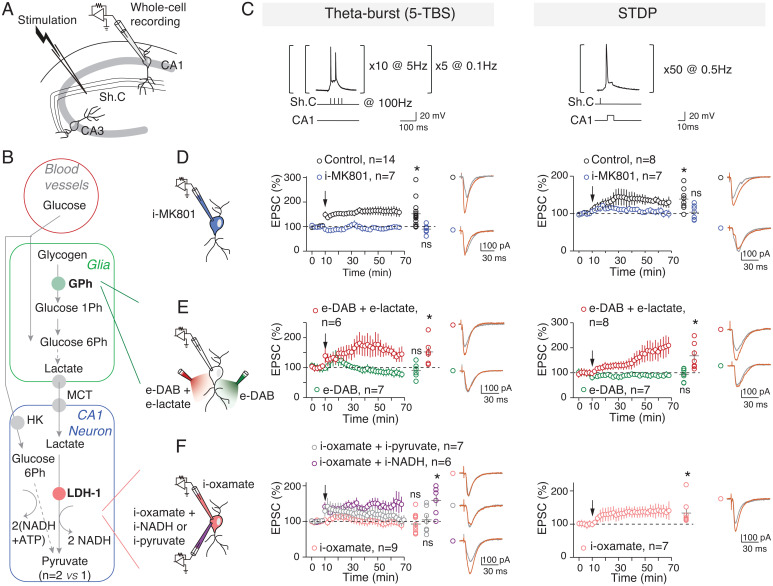
5-TBS–LTP and STDP-LTP rely differently on neuronal lactate. (*A*) Experimental setup. Sh. C, Schaffer collaterals. (*B*) Key steps of glucose transport and glia–neuron lactate transport: glycogen catalysis into glucose-1-phosphate via glycogen phosphorylase, lactate entry in neurons via monocarboxylate transporters (MCTs), and lactate conversion to pyruvate by LDH; Ph, phosphate. (*C*) 5-TBS (with nested high-frequency, 100-Hz stimulations within slower-frequency, 5-Hz stimulations repeated five times at 0.1 Hz) and STDP (50 paired pre/post stimulations at 0.5 Hz) paradigms. (*D*–*G*) Averaged time-course of the synaptic weight with EPSC amplitude 50–60 min after TBS or STDP paradigm. (*D*) 5-TBS–LTP and STDP-LTP (5-TBS–LTP: *P* = 0056, *n* = 14; STDP-LTP: *P* = 0.0070, *n* = 8) were NMDAR mediated (with intracellular application of i-MK801: 5-TBS, *P* = 0.1562, *n* = 7; STDP, *P* = 0.6484, *n* = 7). (*E*) Inhibition of glycogen phosphorylase with bath-applied DAB (e-DAB) prevented 5-TBS–LTP and STDP-LTP (with e-DAB: 5-TBS, *P* = 0.3691, *n* = 6; STDP, *P* = 0.4213, *n* = 7). Coapplication of DAB and lactate allowed 5-TBS–LTP and STDP-LTP (*P* = 0.0311, *n* = 6 and *P* = 0.0383, *n* = 7, respectively). LTP rescued with e-lactate was not significantly different from control (5-TBS-LTP: *P* = 0.6735; STDP-LTP: *P* = 0.4155). (*F*) Intracellular inhibition of LDH revealed distinct effects on 5-TBS and STDP expression because i-oxamate prevented 5-TBS–LTP (*P* = 0.4600, *n* = 9) but not STDP-LTP (*P* = 0.0398, *n* = 7). 5-TBS–LTP was rescued with coapplication of i-oxamate and i-NADH (*P* = 0.0145, *n* = 6 versus 5-TBS–LTP control: *P* = 0.7995) but not i-pyruvate (*P* = 0.7548, *n* = 7). Representative traces show 15 EPSCs averaged during baseline (gray) and 45 min (red) after protocols (arrows). All data are shown as mean ± SEM; *, *P* < 0.05; ns, not significant by two tailed *t* test. See *SI Appendix*, Table S1, for detailed data and statistics.

To investigate the relative involvement of glucose and lactate metabolism in synaptic plasticity, we tested two activity-dependent forms of LTP at hippocampal CA1 pyramidal cells (Fig. 1 *A*–*C*), aiming to reflect two levels of neural computation. We chose two distinct Hebbian activity patterns: 1) a rate-coding paradigm involving a high stimulation load (200 stimulations; 5× theta-burst stimulation [5-TBS] with nested high-frequency [100 Hz] stimulations within slower frequencies [5 Hz] repeated five times [at 0.1 Hz]) and 2) a time-coding paradigm involving a lower stimulation load (50 stimulations; spike timing–dependent plasticity (STDP) with 50 pre- and postsynaptic paired stimulations at a low frequency [0.5 Hz]) (Fig. 1 and *SI Appendix*, Table S1). Whole-cell recordings of CA1 pyramidal cells were performed at a physiological glucose concentration (5 mM) (29) to avoid saturated nonphysiological concentrations of glucose (∼15 to 25 mM) classically used in brain slice studies. In the following experiments, drugs were applied intracellularly (i-drug) via patch-clamp pipette, ensuring specific effects in the sole recorded neuron except in few cases where drugs were bath applied (e-drug). The 5-TBS and STDP paradigms induced LTP, and both were *N*-methyl-d-aspartic acid receptor (NMDAR) mediated because they were prevented by the intracellular application of the NMDAR blocker MK801 (i-MK801; 1 mM) (Fig. 1*D* and *SI Appendix*, Fig. S1). Because both LTP forms share the same signaling pathway, we could interpret their respective glucose/lactate dependency based on the activity patterns. We evaluated whether 5-TBS–LTP and STDP-LTP expression equally relies on lactate metabolism by sequentially inhibiting two key steps: glycogen mobilization into glucose-1-phosphate, the first step of glycogenolysis leading ultimately to glia-derived lactate, via glycogen phosphorylase and conversion of lactate into pyruvate via the neuronal lactate dehydrogenase (LDH-1) (Fig. 1*B*). When glial glycogenolysis was prevented by inhibiting glycogen phosphorylase with 1,4-dideoxy-1,4-imino-d-arabinitol (e-DAB; 10 μM), 5-TBS or STDP pairings failed to induce synaptic plasticity (Fig. 1*E*). We tested whether lactate overcomes DAB effects. With e-DAB and e-lactate (10 mM), we observed 5-TBS–LTP and STDP-LTP (Fig. 1*E*); LTP rescued with e-lactate was not significantly different from controls. This indicates that lactate formed from glycogenolysis is a key factor for hippocampal LTP induction (14, 16, 20). We next prevented the conversion of lactate into pyruvate by applying oxamate (an inhibitor of LDH) intracellularly (i-oxamate; 6 mM) only in the recorded neuron. Under this condition, 5-TBS did not evoke plasticity, whereas STDP-LTP could still be observed (Fig. 1*F*). Conversion of lactate into pyruvate was thus required for 5-TBS–LTP but not for STDP-LTP. We then tested which products of lactate conversion by LDH-1, that is, pyruvate or NADH, was needed for 5-TBS–LTP. When i-pyruvate (10 mM) was coapplied intracellularly with i-oxamate, 5-TBS did not induce plasticity, whereas 5-TBS–LTP was rescued with i-NADH (4 mM) (Fig. 1*F*).

As revealed by LDH inhibition, neuronal lactate appears as a key element for 5-TBS–LTP (via its metabolism to pyruvate leading to NADH production), but is not necessary for inducing a lighter form of activity-dependent plasticity involving 50 STDP pairings, which relies on glucose metabolism when lactate conversion is blocked.

### Confronting Mathematical Model and Experimental Data Delineates the Energetic Needs of Synaptic Plasticity.

**Fig. 2.**
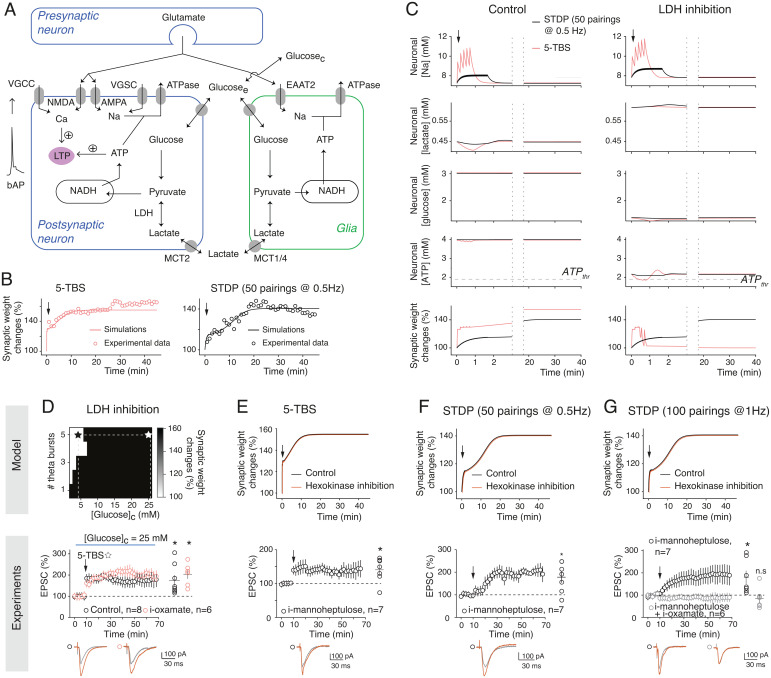
Confronting mathematical model and experimental data delineates the energetic needs of synaptic plasticity. (*A*) In the model (M*aterials and M*ethods and *SI Appendix*, *Supplementary Information for the Mathematical Model, Supplementary Methods and Materials*, and *SI Appendix*, Table S2), the synaptic weight is gated by both neuronal calcium (potentiation) and ATP (depression); VGCC, voltage-gated calcium channel; VGSC, voltage-gated sodium channel; EAAT2, excitatory amino acid transporter-2; AMPA, α-amino-3-hydroxy-5-methyl-4-isoxazolepropionic acid. (*B*) Time course of the synaptic weight in the model (lines) fitted to 5-TBS (*n* = 7) and STDP (*n* = 14; 50 pairings at 0.5 Hz; spike timing = 10 ms) experiments (circles). (*C*) Model prediction for the evolution of neuronal concentrations and synaptic weight with 5-TBS (red) or STDP (black). (*D*) TBS-LTP expression depending on glucose concentration ([Glucose]_c_) as predicted by the model. Experimentally, 5-TBS induced LTP (*P* = 0.0299, *n* = 8) at a high glucose concentration (25 mM), and this LTP was not impaired by LDH inhibition (i-oxamate; *P* = 0.0044, *n* = 6). (*E*) 5-TBS–LTP expression with hexokinase inhibition (i-mannoheptulose; *P* = 0.0230, *n* = 7). (*F*) Fifty pairings at 0.5 Hz induced STDP-LTP with i-mannoheptulose (*P* = 0.0210, *n* = 7). (*G*) One hundred pairings at 1 Hz induced STDP-LTP with i-mannoheptulose (*P* = 0.0436, *n* = 6). When neuronal glycolysis and lactate conversion into pyruvate were inhibited with coapplied i-mannoheptulose and i-oxamate, 100 pairings did not induce plasticity (*P* = 0.5297, *n* = 6). Representative traces show 15 EPSCs averaged during baseline (gray) and 45 min (red) after protocols (arrows). All data are represented as mean ± SEM (except in *B* where SEM was omitted for clarity); *, *P* < 0.05; ns, not significant by two tailed *t* test. See *SI Appendix*, Table S3 for detailed data and statistics.

**Fig. 3.**
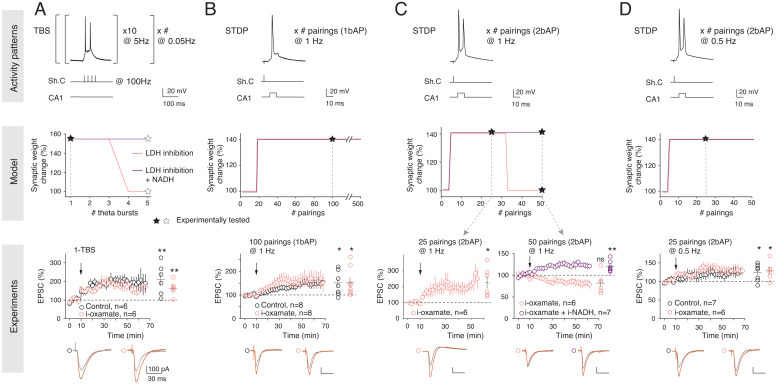
Dependence on lactate for LTP expression is activity pattern linked. (*A*) Lactate-dependent TBS-LTP depends on the number of TBS (*Left*, protocol). The model predicts that TBS-LTP is dependent on the lactate pathway from 4-TBS, as demonstrated experimentally with LTP induced by 1-TBS (*P* = 0.0229, *n* = 6), which was not impaired by i-oxamate (*P* = 0.0109, *n* = 6). (*B*) STDP-LTP induced by 20 to 500 pre/post pairings at 1 Hz with a single bAP/pairing can be induced under LDH inhibition as predicted by the model and demonstrated experimentally with LTP induced by 100 pairings at 1 Hz under control (*P* = 0.0191, *n* = 8) or LDH inhibition (*P* = 0.0391, *n* = 8) conditions, with similar magnitude (*P* = 0.9836). (*C*) STDP-LTP induced by pairings at 1 Hz with two bAPs/pairing is dependent on LDH activity from 30 pairings, as predicted by the model and demonstrated experimentally with i-oxamate, which prevented LTP expression by 50 (*P* = 0.0956, *n* = 6), but not 25 (*P* = 0.0216, *n* = 6), pairings with two bAPs. Intracellular coapplication of i-oxamate and i-NADH rescued STDP-LTP (*P* = 0.0061, *n* = 7), as predicted by the model. (*D*) STDP-LTP induced by pairings at 0.5 Hz with two bAPs/pairing (*P* = 0.0369, *n* = 7) can be induced under LDH inhibition (*P* = 0.0309, *n* = 6), as predicted by the model and demonstrated experimentally with LTP induced by 25 pairings with two bAPs/pairing, which is not impaired with i-oxamate. Black stars indicate the experimental conditions tested. Representative traces show 15 EPSCs averaged during baseline (gray) and 45 min (red) after protocol (arrows). All data are represented as mean ± SEM; *, *P* < 0.05; ns, not significant by two tailed *t* test. See *SI Appendix*, Table S3 for detailed data and statistics.

To provide hypotheses for the differential effects of the neuronal glucose and lactate metabolism on activity-dependent plasticity, we developed a mathematical model of CA1 synaptic plasticity combined with metabolism. Our model describes the kinetics of signaling and metabolic reactions occurring in a neuro–glia unit in response to activity patterns (Fig. 2*A*). The postsynaptic weight is modeled as a bistable system gated by calcium and ATP; postsynaptic calcium triggers LTP when it overcomes a threshold ($LTP_{\text{Start}}$), while the postsynaptic ATP level triggers depotentiation when falling below a second threshold ($ATP_{\text{Thr}}$). Calcium influx in the postsynaptic neuron changes in response to presynaptic and postsynaptic spikes via the activation of NMDAR and voltage-gated calcium channels. ATP levels in each compartment are computed by a model of metabolic interactions with the astrocyte–neuron lactate shuttle (30) that includes, among others, glycolysis and LDH activity in glia and the postsynapse as well as glucose and lactate transfer from glia to neurons via the extracellular medium (*SI Appendix*, *Supplementary Materials and Methods and Supplementary Information for the Mathematical Model*). Importantly, the values of the model parameters were estimated using a subset of our experimental data taken from Figs. 1 *D*–*G* and 2*B* and Tables S1–S3, while model validation was performed using model predictions, that is, by checking the accuracy of the model output under experimental conditions that were not used for parameter estimation (the pharmacological experiments of Figs. 2 *D*–*G* and 3). The model captures the amplitude and kinetics of change of the synaptic weight after 5-TBS and STDP pairings (Fig. 2*B*). In the model, both 5-TBS and STDP paradigms are strong enough to generate large calcium transients in the postsynaptic neuron (*SI Appendix*, Fig. S2*A*) that overcome the $LTP_{\text{Start}}$ threshold, thus triggering LTP. The amplitudes of Na^+^ transients in the postsynaptic neuron are much larger with 5-TBS than with STDP so that ATP consumption by Na,K^+^-ATPases is larger with 5-TBS (Fig. 2*C*). With the model, the concept that different levels of activity pattern loads are engaged in STDP (50 pairings at 0.5 Hz) versus 5-TBS was supported by estimating the neuronal sodium and calcium influxes (that were <1 versus >3 mM and ∼0.75 versus ∼1.5 μM, respectively) or the amplitude of ATP consumption used for sodium extrusion (∼90 versus ∼300 μM; Fig. 2*C* and *SI Appendix*, Fig. S2*A*). The availability of lactate as a source of ATP keeps ATP levels well above $ATP_{\text{Thr}}$ even after 5-TBS. LDH inhibition switches the neuron to a glycolytic regimen, an oxidized redox state where ATP level drops to 2.1 mM at rest (*SI Appendix*, Fig. S3). After STDP and with LDH inhibition, the ATP levels keep well above $ATP_{\text{Thr}}$, while with 5-TBS, ATP quickly fails below $ATP_{\text{Thr}}$, and the resulting depotentiation forbids LTP expression (Fig. 2*C*). Experimentally, we found that pyramidal cells recorded with lower i-ATP and i-phosphocreatine (2 and 5 mM, respectively) did not exhibit plasticity following STDP (50 pairings at 0.5 Hz) in control or i-oxamate (*SI Appendix*, Fig. S4).

Using a model-guided approach, we investigated the impact of glucose on LTP expression. Fig. 2*D* shows model output for TBS with LDH inhibited depending on extracellular glucose concentration. The model predicts that LTP recovers if bath glucose concentration is large enough. Experiments confirmed that with high glucose concentration (25 mM), 5-TBS–LTP was no longer sensitive to i-oxamate (Fig. 2*D*). Another model prediction is that the hexokinase inhibition, the first enzyme of glycolysis catalyzing the phosphorylation of glucose to glucose-6-phosphate (Fig. 1*B*), should not affect 5-TBS–LTP (Fig. 2*E*). Indeed, experimentally, a specific inhibitor of the hexokinase, mannoheptulose, applied intracellularly (i-mannoheptulose; 10 μM) did not prevent 5-TBS–LTP (Fig. 2*E*), confirming that 5-TBS–LTP relies on lactate and not on glucose metabolism. We next explored the glucose dependency of STDP, and, as predicted by the model, we found that i-mannoheptulose did not prevent STDP-LTP (Fig. 2*F*), indicating that in the absence of neuronal glycolysis, the lactate pathway is used for the expression of STDP-LTP (50 pairings at 0.5 Hz). We next doubled the number and frequency of STDP pairings (up to 100 pairings at 1 Hz) to test whether this property could be extended to other STDP forms. In confirmation of the model prediction, we found that 100 pairings at 1 Hz induced LTP with i-mannoheptulose (Fig. 2*G*). Interestingly, when both neuronal glucose and lactate sources were impaired by the intracellular coapplication of i-mannoheptulose and i-oxamate, (100 pairings at 1 Hz) STDP-LTP was not observed (Fig. 2*G*), showing that STDP relies on either glycolysis or lactate pathway. Together, these model-guided experiments show that TBS-LTP relies on lactate, although high glucose can bypass lactate fueling, whereas STDP can use either the glycolysis or lactate pathways.

### Dependence on Glucose versus Lactate for LTP Expression Is Activity Pattern Linked.

We varied the TBS and STDP activity patterns to delineate the sensitivity of the plasticity dependency on glucose and lactate metabolism. Model calibration relied exclusively on 5-TBS and STDP with 50 pairings at 0.5 Hz (Fig. 2*B*). We first generated model predictions by varying the number of TBS bursts and predicted that LTP induction by one single burst (1-TBS) would not be dependent on lactate metabolism, whereas LTP induced with at least four bursts (4-TBS) would be, unless performed with high levels of added NADH (Fig. 3*A*). This was validated experimentally because 1-TBS–LTP could be induced with i-oxamate (Fig. 3*A*). As for predictions related to STDP, we first varied in silico the number of pairings and predicted that STDP, even for 500 pairings, would remain nondependent on lactate metabolism (Fig. 3*B*). This was demonstrated experimentally because 100 pairings with a single back-propagating action potential (bAP) at 1 Hz induced LTP in control and with i-oxamate with similar magnitude (Fig. 3*B* and *SI Appendix*, Table S3). This was confirmed in mice where i-oxamate prevented 5-TBS–LTP but not LTP induced by STDP with 100 pairings (*SI Appendix*, Fig. S5).

We next varied the number of bAPs per STDP pairings and tested the impact of one additional bAP, that is, going from one to two bAPs per STDP pairing. Two-photon imaging of dendritic spines and shafts of CA1 pyramidal cells showed that the calcium transient triggered by two bAPs was roughly twofold compared to one bAP (*SI Appendix*, Fig. S6), a feature reproduced by the model (*SI Appendix*, Fig. S2*B*). For plasticity induction, we kept constant the overall number of postsynaptic stimulations, maintaining a total of 100 pairings (100 pairings with one bAP versus 50 pairings with two bAPs) and the same frequency (1 Hz). The model predicted that shifting from one to two bAPs would render STDP lactate dependent if more than 30 pairings were used (Fig. 3*C*).

Experimentally, with i-oxamate, LTP was induced by 25 pairings at 1 Hz with two bAPs, while no plasticity was detected for 50 pairings (with two bAPs), and adding i-NADH to i-oxamate rescued LTP expression, in agreement with model prediction (Fig. 3*C*). These observations illustrate that the on-demand fueling is highly sensitive to the activity patterns on either side of the synapse because a variation from one to two bAPs was sufficient to render the lactate pathway necessary for LTP expression.

We finally tested whether the number of bAPs per se or its combination with the number and frequency of pairings matters. To do so, we kept two bAPs per pairing and decreased the number and frequency of pairings twofold to compare this condition with the STDP paradigm used in Fig. 1, that is, 50 postsynaptic stimulations overall (50 pairings with one bAP versus 25 pairings with two bAPs, both at 0.5 Hz) (Fig. 3*D*). The model predicted that under these conditions, LTP would be induced and would not depend on lactate. Experimentally, we found that 25 pairings with two bAPs (at 0.5 Hz) induced LTP, and that this LTP was still observed with i-oxamate (Fig. 3*D*). Therefore, the dependence on glucose versus lactate metabolism precisely scales with the activity patterns used to induce plasticity; glia-derived lactate is required for sustained activity-dependent plasticity, and neuronal glycolysis is sufficient for plasticity induced by lower synaptic activity.

### Inhibition of LDH Impairs OiP but not NOR Learning.

**Fig. 4.**
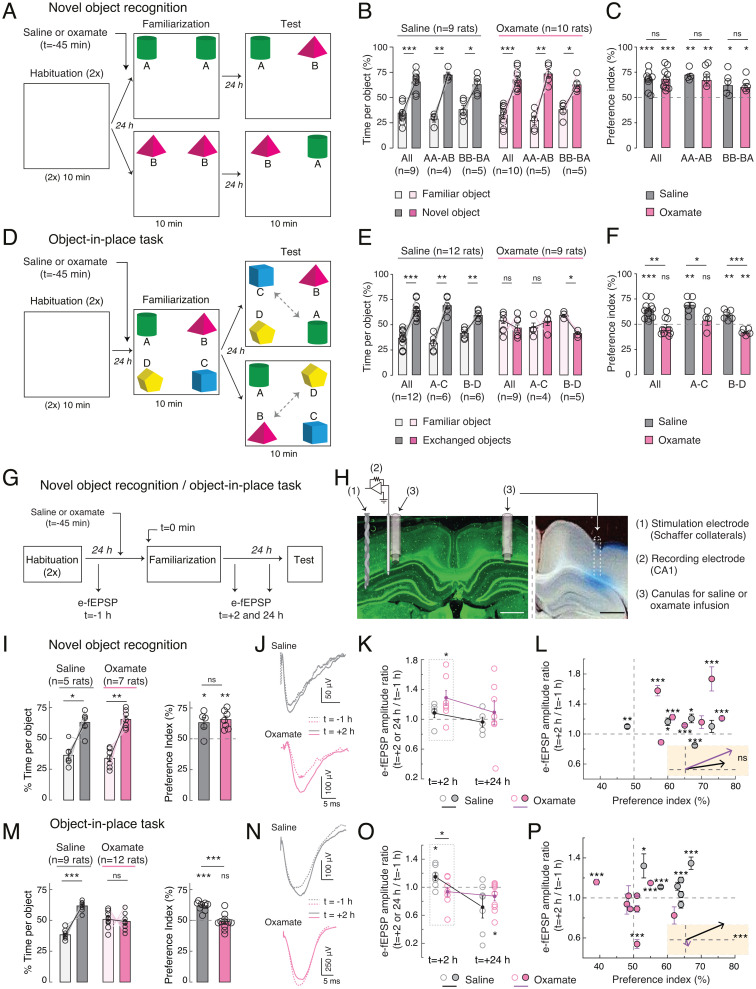
Inhibition of LDH impairs OiP and associated LTP but not NOR learning. (*A*–*F*) NOR and OiP tasks were similarly structured into three phases conducted at a 1-d interval: 1) habituation phase in the empty arena, 2) familiarization phase in the presence of two (NOR) or four (OiP) objects, and 3) test in which recognition of new (NOR) or exchanged objects (OiP) was assessed. Rats were injected bilaterally via cannulas implanted just above the CA1 layer with saline or oxamate (50 mM) solutions 45 min before starting the familiarization phase. (*A*–*C*) NOR task with two objects. (*A*) Experimental setup. Rats were divided in two subgroups exposed to A-A and then A-B or B-B and then B-A during familiarization and test phases, respectively. (*B* and *C*) Rats injected in the CA1 with saline or oxamate (50 mM) spent equally more time exploring the novel object (saline: *P* = 0.0007, *n* = 9; oxamate: *P* = 0.0003, *n* = 10; saline versus oxamate: *P* = 0.8126) as assessed by the time per object (*B*) and the preference index (*C*). LDH inhibition did not impair novelty detection. (*D*–*F*) OiP task with four objects. (*D*) Rats were exposed to A-B-C-D objects during familiarization and were divided in two subgroups experimenting A-C or B-D exchanged-object for test. (*E* and *F*) Saline-injected rats spent more time exploring the exchanged objects, whereas oxamate-injected rats explored equally all objects (saline: *P* < 0.0001, *n* = 12; oxamate: *P* = 0.2500, *n* = 9; saline versus oxamate: *P* = 0.0002). LDH inhibition impaired the ability of rats to detect place-exchanged objects. (*G*–*P*) e-fEPSP recordings during NOR and OiP tasks. (*G*) Experimental setup. (*H*) Microphotographs showing cannulas and stimulation/recording electrode locations and diffusion area. (Scale bars, 1 mm.) Chronic stimulating and recording electrodes were placed in Schaffer collaterals and the CA1, respectively, in rats equipped bilaterally with cannulas for saline or oxamate infusion. (*I*–*P*) In vivo synaptic plasticity during NOR and OiP. e-fEPSPs were recorded before familiarization (baseline) and 2 and 24 h after familiarization to determine synaptic changes in relation to behavior. (*I–L*) NOR behavioral performance (*I*) with related e-fEPSPs showing LTP after 2 h but not after 24 h in saline- and oxamate-injected rats (*J*–*L*); averaged vectors show similar trends (*P* = 0.412) (*L*). (*M*–*P*) OiP behavioral performance (*M*) with related e-fEPSPs show LTP in saline-injected but not in oxamate-injected rats 2 h after familiarization (*N*–*P*); averaged vectors show differences between saline- and oxamate-injected rats (*P* < 0.001) (*P*). All data represent mean ± SEM; *, *P* < 0.05; **, *P* < 0.01; ***, *P* < 0.001; data were analyzed by two-tailed *t* test. See *SI Appendix*, Table S4 *A–D* for detailed data and statistics.

Because synaptic plasticity is a major substrate for learning and memory (31), we next tested whether lactate dependency scales with learning of recognition memory tasks with increasing cognitive loads. For this purpose, we chose two single-trial tasks involving the hippocampus and perirhinal cortex, which differ by their difficulty level; the OiP task (with four objects) is more challenging and requires a higher cognitive load than the NOR task (with two objects). These tasks were similarly structured into three phases conducted at a 1-d interval: 1) habituation phase in the empty arena, 2) familiarization phase in the presence of two (NOR) or four (OiP) objects, and 3) test in which recognition of new (NOR) or exchanged objects (OiP) was assessed. Rats were injected bilaterally via cannulas implanted just above the CA1 layer with saline or oxamate (50 mM) solutions 45 min before starting the familiarization phase (Fig. 4).

For NOR with two objects (A and B), rats injected with saline (*n* = 9) or oxamate (*n* = 10) performed similarly (Fig. 4 *A*–*C*). Indeed, rats spent more time during the test phase around the new object when considering the time per object and the preference index. We ensured that there was no significant preference for object A or B during familiarization because rats (injected in the CA1 with saline or oxamate solutions) spent similar amounts of time exploring objects A and B (*SI Appendix*, Fig. S7*A*). Similar results were found regardless of whether the familiarization and test phases were achieved with AA/AB (*n* = 4 and 5 rats in saline and oxamate, respectively) or BB/BA (*n* = 5 and 5 rats in saline and oxamate, respectively) object combination (Fig. 4 *A*–*C* and *SI Appendix*, Table S4*A*). Indeed, rats detected novelty under saline and oxamate conditions as indicated by the time per object (Fig. 4*B*) and by preference index (Fig. 4*C*) in both AA/AB and BB/BA object combinations.

We next subjected rats to the OiP task (Fig. 4 *D–F*): saline- or oxamate-injected rats (*n* = 12 and 9, respectively) were left for familiarization with four different objects (A, B, C, and D) for which they did not show preference (*SI Appendix*, Fig. S7*B*), and then for the test phase, two of the four objects were place exchanged (from ABCD to CBAD or to ADCB). We found that rats injected with saline spent more time around the exchanged objects, whereas rats injected with oxamate did not notice the exchange because they continued exploring equally the four objects (Fig. 4 *D*–*F* and *SI Appendix*, Table S4*B*). Similar results were found regardless of whether the familiarization and test phases were performed with ABCD/CBAD (*n* = 6 and 4 rats in saline and oxamate, respectively) or ABCD/ADCB (*n* = 6 and 5 rats in saline and oxamate, respectively) object exchange combination.

Therefore, when conversion of lactate into pyruvate was impaired during familiarization, rats still succeeded in NOR but not in the more challenging OiP task.

### Inhibition of LDH Impairs OiP-Induced LTP but not NOR-Induced LTP.

We further examined in NOR and OiP tasks whether synaptic plasticity occurred with CA1 hippocampal infusion of saline or oxamate in relation to behavioral performance (Fig. 4 *G*–*P* and *SI Appendix*, Fig. S7 *C–G* and Tables S4 *A–D*). Synaptic weights were evaluated in vivo by monitoring evoked-field excitatory postsynaptic potentials (e-fEPSPs) at synapses between Schaffer collaterals and CA1 pyramidal cells in behaving rats. To do so, we placed chronic stimulating and recording electrodes in Schaffer collaterals and CA1, respectively, in rats equipped bilaterally with cannulas for saline or oxamate infusion (Fig. 4*H*). We first ensured that this set of rats performed in NOR and OiP similarly as mentioned earlier. In detail, rats infused with saline (*n* = 5) or oxamate (*n* = 7) detected novelty in NOR task (AA-AB) (Fig. 4*I* and *SI Appendix*, Table S4*A*). Regarding the OiP task, rats subjected to saline (*n* = 9) were able to detect novelty, whereas rats infused with oxamate (*n* = 12) did not (ABCD/ADCB) (Fig. 4*M* and *SI Appendix*, Table S4*B*). To determine plasticity expression, e-fEPSPs were monitored before (*t* = −1 h before saline or oxamate injection) and after (*t* = +2 and +24 h) the familiarization phase (Fig. 4 *J*–*P* and *SI Appendix*, Fig. S7 *C–G*).

In the NOR task, LTP of e-fEPSPs dominated at *t* = +2 h in both saline- and oxamate-injected rats (*n* = 5 and 7, respectively) and is followed by a scaled reduction of synaptic weights at *t* = +24 h (as shown by the positive correlation between plasticities at *t* = +2 and +24 h), leading, on average, to no plasticity at *t* = +24 h (Fig. 4 *J* and *L* and *SI Appendix*, Fig. S7 *C–G* and Table S4*C*). The behavioral and plasticity profiles were similar between saline- and oxamate-injected rats, as illustrated by the average vectors considering the preference index and plasticity at *t* = +2 h after familiarization (Fig. 4*L* and *SI Appendix*, Fig. S7*F* and Table S4*D*).

A different picture was obtained in the OiP task. e-fEPSPs exhibited LTP at *t* = +2 h in saline-injected rats but not in oxamate-injected rats (Fig. 4*O*). More precisely, when considering e-fEPSP plasticity at 2 h after familiarization in relation to behavioral performance, all saline-injected rats detected exchanged objects, and four of seven displayed LTP, whereas among the 78% (seven of nine) of the oxamate-injected rats that did not detect the exchange, only one showed LTP, while the others exhibited an absence of plasticity or long-term depression (LTD). This is also illustrated by the difference between averaged vectors (Fig. 4*P* and *SI Appendix*, Table S4*D*). Monitoring of the synaptic weights 24 h after familiarization showed similar plasticity pictures for saline- and oxamate-injected rats (*n* = 9 and 12, respectively), that is, LTD or the absence of plasticity despite distinct preference indexes (*SI Appendix*, Fig. S7 *E* and *G* and Table S4*D*).

In conclusion, rats detecting novelty in the OiP task displayed LTP after the familiarization phase, whereas oxamate-injected rats, which were not able to detect novelty, did not show LTP. Therefore, learning novelty in a challenging memory task (OiP) requires lactate-dependent LTP, while glucose-dependent LTP can be sufficient to learn a less demanding cognitive task (NOR).

## Discussion

Here, we show that scaling of computational and cognitive loads requires the metabolism of astrocytic glycogen-derived lactate to match the energetic requirements of sustained neural activity patterns and high cognitive load. For less demanding plasticity and learning paradigms, glucose suffices as an energy substrate. We thus reconcile conflicting views concerning the involvement of lactate versus glucose in synaptic plasticity (11, 26, 27, 32). The two pools of energy substrates (glucose and lactate) can be distinctly allocated on demand (33–37) in qualitative (activity hotspots) and quantitative (engram levels) manners within the hard limit of the global energy availability of cellular metabolism (38, 39). We delineated the domains of activity pattern for which LTP expression requires glucose and/or lactate metabolism and their borders defined by the elementary elements of neural computation, that is, the rate and timing codes. This is particularly illustrated by the fact that variation of a single bAP was sufficient to shift the LTP dependency from glucose to lactate.

Physiologically, lactate in the brain can be formed through two metabolic pathways that correspond to two forms of the astrocyte–neuron lactate shuttle, glycogenolysis and glutamate-stimulated glucose uptake into astrocytes (7, 11). Glycogenolysis in astrocytes is promoted by neuromodulators, such as noradrenaline and vasoactive intestinal peptide (7, 23). The glia-derived lactate, as well as neuronal glycolysis, could thus be triggered after extracellular potassium changes as low as ∼200 µM, according to theoretical estimations of the potassium efflux upon a single action potential (40), consistent with the demonstrated glycogenolytic action of potassium (41–43). Interestingly, noradrenaline is released from fibers in the cortex and hippocampus during task-relevant stimuli, optimizing behavioral performance (44). The firing of the locus coeruleus, where noradrenaline-containing cell bodies are localized, renders neurons in the cortex and hippocampus more responsive to a broad spectrum of stimuli, including behavioral attentional states (44, 45) involved in plasticity and learning. In the context of the observations presented here, it is worth noting that glycogenolysis triggered by activation of beta2 receptors selectively localized on astrocytes is necessary for memory consolidation (16).

Increased energetic demands on astrocytes such as glutamate uptake, one of the main functions of astrocytes, stimulates glucose uptake and the activation of its metabolism through aerobic glycolysis, resulting in lactate production (10). One may therefore wonder which one of the two forms of the astrocyte–neuron lactate shuttle is mobilized during the higher energy-demanding plasticity loads described in this article. The role of glycogen-derived lactate in memory consolidation is now well established (14–16). Recent modeling data show that the glycogenolysis-derived lactate evoked by a glycogenolytic neuromodulator such as noradrenaline (23) operates with much shorter time constants than the glutamate uptake–triggered one (46).

The demonstration that under conditions of glycogenolysis inhibition, lactate, but not glucose, allows sustained electrical activity (9), fear, or spatial learning (14–16) (that involves high perceptual load) is in line with our results. Also, a decrease in lactate production mediated by mitochondrial cannabinoid type-1 receptor activation in astrocytes alters social behavior in a lactate-reversible manner (47). By contrast, fast-learning engrams originating from light activity patterns (48) could emerge even in the absence of lactate metabolism, with glucose as the main energy substrate.

Our mathematical model predicts that, with oxamate, NADH levels during TBS are larger than during STDP, although less than in control conditions, that is, without LDH inhibition: NADH(STDP + oxamate) < NADH(TBS + oxamate) < NADH(TBS or STDP in control). Because TBS + oxamate is the only condition where LTP is suppressed, we concluded that a simple control of LTP by the level of NADH is unlikely. The dynamics of cytosolic ATP levels were more compatible with a simple gating mechanism because ATP levels in the model were found especially low with TBS + oxamate and much larger for STDP + oxamate or under control conditions (TBS or STDP without oxamate). ATP is usually believed to be tightly regulated in neurons (2, 3, 7), so our model prediction that oxamate causes a twofold decrease of cytosolic ATP at rest is unexpected. We cannot rule out that the metabolite that limits TBS-LTP with oxamate is not ATP but one of its less tightly regulated metabolites or binding partners and one that the model would not account for (ATPases or other ATP-dependent enzymes or channels, for instance). Continuous measurements of cytosolic neuronal ATP/ADP and NADH/NAD^+^ ratios during TBS or STDP, with and without oxamate, would allow testing our hypothesis. However, to our knowledge, single-cell ATP monitoring in neurons during LTP protocols is still an experimental challenge in brain slices. In any case, the model prediction of depotentiation when ATP drops below a threshold (Fig. 2*C*) provides a simple mechanism to link metabolism with the signaling pathways of synaptic plasticity. This simple mechanism endowed the model with strong predictive properties, allowing us to correctly forecast the results of a wide range of experimental conditions (number or frequencies of action potentials and glucose concentration). This advocates in favor of the validity of our hypothesis of an ATP-gated depotentiation.

The tight dependence and sensitivity of neuronal signaling on energy availability renders the brain vulnerable to conditions in which energy delivery or utilization are compromised. This is the case for neurodegenerative diseases, such as Alzheimer’s and Parkinson’s diseases, amyotrophic lateral sclerosis, and frontotemporal dementia (49–52), as well as for neurodevelopmental disorders, such as glucose transporter-1 deficiency syndrome (53). Pharmacological strategies aimed at boosting brain energy metabolism by acting at specific cellular and molecular targets (e.g., neurons versus glial cells and glycolysis versus glycogenolysis) deserve close attention, as they may provide an original and unifying interventional approach for diseases characterized by cognitive impairment and neurodegeneration.

## Materials and Methods

Detailed materials and methods, that is, patch-clamp whole-cell and two-photon recordings in brain slices, behavioral tasks, in vivo electrophysiological recordings in behaving rats, and mathematical models, are included in the *SI Appendix*, *Supplementary Information for the Mathematical Model* and *Supplementary Materials and Methods*.

Experiments were conducted in male Sprague–Dawley rats (Charles River) P_30 to 35 d_ for brain slice patch-clamp and two-photon imaging and P_7 to 9 wk_ for behavioral tasks and in vivo electrophysiology and in C57BL/6 mice P_28–35 d_ for brain slice electrophysiology (*SI Appendix*, Fig. S5).

### Whole-Cell CA1 Pyramidal Neuron Recordings.

For whole-cell CA1 pyramidal neuron recordings, transverse hippocampal slices (350 µm thick) were prepared. Signals were amplified with EPC10-2 amplifiers (HEKA Elektronik). Current- and voltage-clamp recordings were sampled at 20 kHz with the Patchmaster v2 × 32 program (HEKA Elektronik). All recordings were performed at 35 °C.

### Synaptic Plasticity Induction Protocols.

Synaptic responses in CA1 pyramidal cells were evoked by electrical stimulations of Schaeffer’s collaterals with concentric bipolar electrodes (Phymep) placed in the stratum radiatum area of the hippocampus, with two paradigms being applied, TBS and STDP.

### NOR and OiP tasks.

The NOR (two objects) and OiP (four objects) tasks involved three sessions on three consecutive days: habituation (on day 1), familiarization (on day 2), and test (on day 3).

### In Vivo Electrophysiology in Behaving Rats during the NOR and OiP Tasks.

fEPSPs evoked from Schaffer collateral stimulation (e-fEPSPs) were measured (KJE-1001 system, Amplipex) in the left CA1 over the 3-d behavioral assessment in rats subjected to NOR or OiP tasks.

### Mathematical Model.

Our model simulates the network of signaling and metabolic reactions occurring in a postsynaptic neuronal terminal and an interacting astrocyte, as shown in Fig. 2*A*. The postsynaptic weight was modelled using a bistable ordinary differential equation where potentiation is triggered by high intracellular calcium whereas low cytosolic ATP levels cause depotentiation. The level of intracellular calcium depended on the set of channels and pumps illustrated in Fig. 2*A*, whereas the time course of ATP concentration was estimated using a model of neuron-glia metabolic interactions (30), thus effectively linking metabolism and plasticity. For parameter values of the mathematical model, see *SI Appendix*, Table S2.

## Supplementary Material

Supplementary File

## Data Availability

Computer code for the model is publicly available at GitLab (https://gitlab.inria.fr/hberry/anls_stdp) (54). All other study data are included in the article and/or *SI Appendix*.

## References

[1] E. Bullmore, O. Sporns, The economy of brain network organization. Nat. Rev. Neurosci. 13, 336–349 (2012). 22498897 10.1038/nrn3214

[2] J. J. Harris, R. Jolivet, D. Attwell, Synaptic energy use and supply. Neuron 75, 762–777 (2012). 22958818 10.1016/j.neuron.2012.08.019

[3] S. Li, Z.-H. Sheng, Energy matters: Presynaptic metabolism and the maintenance of synaptic transmission. Nat. Rev. Neurosci. 23, 4–22 (2022). 34782781 10.1038/s41583-021-00535-8

[4] K. Mann, S. Deny, S. Ganguli, T. R. Clandinin, Coupling of activity, metabolism and behaviour across the *Drosophila* brain. Nature 593, 244–248 (2021). 33911283 10.1038/s41586-021-03497-0 PMC10544789

[5] D. Tingley, K. McClain, E. Kaya, J. Carpenter, G. Buzsáki, A metabolic function of the hippocampal sharp wave-ripple. Nature 597, 82–86 (2021). 34381214 10.1038/s41586-021-03811-w PMC9214835

[6] C. R. Figley, P. W. Stroman, The role(s) of astrocytes and astrocyte activity in neurometabolism, neurovascular coupling, and the production of functional neuroimaging signals. Eur. J. Neurosci. 33, 577–588 (2011). 21314846 10.1111/j.1460-9568.2010.07584.x

[7] P. J. Magistretti, I. Allaman, Lactate in the brain: From metabolic end-product to signalling molecule. Nat. Rev. Neurosci. 19, 235–249 (2018). 29515192 10.1038/nrn.2018.19

[8] E. R. Zimmer , [^18^F]FDG PET signal is driven by astroglial glutamate transport. Nat. Neurosci. 20, 393–395 (2017). 28135241 10.1038/nn.4492 PMC5378483

[9] A. Trevisiol , Monitoring ATP dynamics in electrically active white matter tracts. eLife 6, e24241 (2017). 28414271 10.7554/eLife.24241 PMC5415357

[10] L. Pellerin, P. J. Magistretti, Glutamate uptake into astrocytes stimulates aerobic glycolysis: A mechanism coupling neuronal activity to glucose utilization. Proc. Natl. Acad. Sci. U.S.A. 91, 10625–10629 (1994). 7938003 10.1073/pnas.91.22.10625 PMC45074

[11] L. F. Barros, B. Weber, CrossTalk proposal: An important astrocyte-to-neuron lactate shuttle couples neuronal activity to glucose utilisation in the brain. J. Physiol. 596, 347–350 (2018). 29292516 10.1113/JP274944 PMC5792514

[12] A. Karagiannis , Lactate is an energy substrate for rodent cortical neurons and enhances their firing activity. eLife 10, e71424 (2021). 34766906 10.7554/eLife.71424 PMC8651295

[13] A. I. Ivanov , Glycolysis and oxidative phosphorylation in neurons and astrocytes during network activity in hippocampal slices. J. Cereb. Blood Flow Metab. 34, 397–407 (2014). 24326389 10.1038/jcbfm.2013.222 PMC3948126

[14] A. Suzuki , Astrocyte–neuron lactate transport is required for long-term memory formation. Cell 144, 810–823 (2011). 21376239 10.1016/j.cell.2011.02.018 PMC3073831

[15] L. A. Newman, D. L. Korol, P. E. Gold, Lactate produced by glycogenolysis in astrocytes regulates memory processing. PLoS One 6, e28427 (2011). 22180782 10.1371/journal.pone.0028427 PMC3236748

[16] C. M. Alberini, E. Cruz, G. Descalzi, B. Bessières, V. Gao, Astrocyte glycogen and lactate: New insights into learning and memory mechanisms. Glia 66, 1244–1262 (2018). 29076603 10.1002/glia.23250 PMC5903986

[17] P. E. Steadman , Disruption of oligodendrogenesis impairs memory consolidation in adult mice. Neuron 105, 150–164 (2020). 31753579 10.1016/j.neuron.2019.10.013 PMC7579726

[18] A. L. Kauffman, J. M. Ashraf, M. R. Corces-Zimmerman, J. N. Landis, C. T. Murphy, Insulin signaling and dietary restriction differentially influence the decline of learning and memory with age. PLoS Biol. 8, e1000372 (2010). 20502519 10.1371/journal.pbio.1000372 PMC2872642

[19] K. D. Longden, T. Muzzu, D. J. Cook, S. R. Schultz, H. G. Krapp, Nutritional state modulates the neural processing of visual motion. Curr. Biol. 24, 890–895 (2014). 24684935 10.1016/j.cub.2014.03.005

[20] C. Murphy-Royal , Stress gates an astrocytic energy reservoir to impair synaptic plasticity. Nat. Commun. 11, 2014 (2020). 32332733 10.1038/s41467-020-15778-9 PMC7181611

[21] M. Zuend , Arousal-induced cortical activity triggers lactate release from astrocytes. Nat. Metab. 2, 179–191 (2020). 32694692 10.1038/s42255-020-0170-4

[22] Z. Padamsey, D. Katsanevaki, N. Dupuy, N. L. Rochefort, Neocortex saves energy by reducing coding precision during food scarcity. Neuron 110, 280–296 (2022). 34741806 10.1016/j.neuron.2021.10.024 PMC8788933

[23] P. J. Magistretti, J. H. Morrison, W. J. Shoemaker, V. Sapin, F. E. Bloom, Vasoactive intestinal polypeptide induces glycogenolysis in mouse cortical slices: A possible regulatory mechanism for the local control of energy metabolism. Proc. Natl. Acad. Sci. U.S.A. 78, 6535–6539 (1981). 6118864 10.1073/pnas.78.10.6535 PMC349075

[24] A. M. Brown, B. R. Ransom, Astrocyte glycogen and brain energy metabolism. Glia 55, 1263–1271 (2007). 17659525 10.1002/glia.20557

[25] J. P. Bolaños, A. Almeida, S. Moncada, Glycolysis: A bioenergetic or a survival pathway? Trends Biochem. Sci. 35, 145–149 (2010). 20006513 10.1016/j.tibs.2009.10.006

[26] C. M. Díaz-García , Neuronal stimulation triggers neuronal glycolysis and not lactate uptake. Cell Metab. 26, 361–374 (2017). 28768175 10.1016/j.cmet.2017.06.021 PMC5559896

[27] G. Yellen, Fueling thought: Management of glycolysis and oxidative phosphorylation in neuronal metabolism. J. Cell Biol. 217, 2235–2246 (2018). 29752396 10.1083/jcb.201803152 PMC6028533

[28] E. de Tredern , Glial glucose fuels the neuronal pentose phosphate pathway for long-term memory. Cell Rep. 36, 109620 (2021). 34433052 10.1016/j.celrep.2021.109620 PMC8411112

[29] R. Gruetter, K. Ugurbil, E. R. Seaquist, Steady-state cerebral glucose concentrations and transport in the human brain. J. Neurochem. 70, 397–408 (1998). 9422387 10.1046/j.1471-4159.1998.70010397.x

[30] R. Jolivet, J. S. Coggan, I. Allaman, P. J. Magistretti, Multi-timescale modeling of activity-dependent metabolic coupling in the neuron–glia–vasculature ensemble. PLoS Comput. Biol. 11, e1004036 (2015). 25719367 10.1371/journal.pcbi.1004036 PMC4342167

[31] S. Nabavi , Engineering a memory with LTD and LTP. Nature 511, 348–352 (2014). 24896183 10.1038/nature13294 PMC4210354

[32] L. K. Bak, A. B. Walls, CrossTalk opposing view: Lack of evidence supporting an astrocyte-to-neuron lactate shuttle coupling neuronal activity to glucose utilisation in the brain. J. Physiol. 596, 351–353 (2018). 29292507 10.1113/JP274945 PMC5792606

[33] K. A. Kasischke, H. D. Vishwasrao, P. J. Fisher, W. R. Zipfel, W. W. Webb, Neural activity triggers neuronal oxidative metabolism followed by astrocytic glycolysis. Science 305, 99–103 (2004). 15232110 10.1126/science.1096485

[34] J. Chuquet, P. Quilichini, E. A. Nimchinsky, G. Buzsáki, Predominant enhancement of glucose uptake in astrocytes versus neurons during activation of the somatosensory cortex. J. Neurosci. 30, 15298–15303 (2010). 21068334 10.1523/JNEUROSCI.0762-10.2010 PMC2997269

[35] P. Mächler , In vivo evidence for a lactate gradient from astrocytes to neurons. Cell Metab. 23, 94–102 (2016). 26698914 10.1016/j.cmet.2015.10.010

[36] I. Ruminot, J. Schmälzle, B. Leyton, L. F. Barros, J. W. Deitmer, Tight coupling of astrocyte energy metabolism to synaptic activity revealed by genetically encoded FRET nanosensors in hippocampal tissue. J. Cereb. Blood Flow Metab. 39, 513–523 (2019). 29083247 10.1177/0271678X17737012 PMC6421254

[37] C. N. Hall, M. C. Klein-Flügge, C. Howarth, D. Attwell, Oxidative phosphorylation, not glycolysis, powers presynaptic and postsynaptic mechanisms underlying brain information processing. J. Neurosci. 32, 8940–8951 (2012). 22745494 10.1523/JNEUROSCI.0026-12.2012 PMC3390246

[38] D. D. Clarke, L. Sokoloff, “Circulation and Energy Metabolism of the Brain” in Basic Neurochemistry: Molecular, Cellular and Medical Aspects, G. J. Siegel , Eds. (Lippincott-Raven, Philadelphia, ed. 6, 1999), vol. 31, chap. 31.

[39] M. Bruckmaier, I. Tachtsidis, P. Phan, N. Lavie, Attention and capacity limits in perception: A cellular metabolism account. J. Neurosci. 40, 6801–6811 (2020). 32747442 10.1523/JNEUROSCI.2368-19.2020 PMC7455219

[40] M. J. Saetra, G. T. Einevoll, G. Halnes, An electrodiffusive neuron-extracellular-glia model for exploring the genesis of slow potentials in the brain. PLOS Comput. Biol. 17, e1008143 (2021). 34270543 10.1371/journal.pcbi.1008143 PMC8318289

[41] P. R. Hof, E. Pascale, P. J. Magistretti, K^+^ at concentrations reached in the extracellular space during neuronal activity promotes a Ca^2+^-dependent glycogen hydrolysis in mouse cerebral cortex. J. Neurosci. 8, 1922–1928 (1988). 3385482 10.1523/JNEUROSCI.08-06-01922.1988 PMC6569342

[42] C. X. Bittner , Fast and reversible stimulation of astrocytic glycolysis by K^+^ and a delayed and persistent effect of glutamate. J. Neurosci. 31, 4709–4713 (2011). 21430169 10.1523/JNEUROSCI.5311-10.2011 PMC6622916

[43] H. B. Choi , Metabolic communication between astrocytes and neurons via bicarbonate-responsive soluble adenylyl cyclase. Neuron 75, 1094–1104 (2012). 22998876 10.1016/j.neuron.2012.08.032 PMC3630998

[44] G. Aston-Jones, J. D. Cohen, An integrative theory of locus coeruleus–norepinephrine function: Adaptive gain and optimal performance. Annu. Rev. Neurosci. 28, 403–450 (2005). 16022602 10.1146/annurev.neuro.28.061604.135709

[45] S. Bouret, S. J. Sara, Reward expectation, orientation of attention and locus coeruleus–medial frontal cortex interplay during learning. Eur. J. Neurosci. 20, 791–802 (2004). 15255989 10.1111/j.1460-9568.2004.03526.x

[46] J. S. Coggan , Norepinephrine stimulates glycogenolysis in astrocytes to fuel neurons with lactate. PLOS Comput. Biol. 14, e1006392 (2018). 30161133 10.1371/journal.pcbi.1006392 PMC6160207

[47] D. Jimenez-Blasco , Glucose metabolism links astroglial mitochondria to cannabinoid effects. Nature 583, 603–608 (2020). 32641832 10.1038/s41586-020-2470-y

[48] C. Piette, J. Touboul, L. Venance, Engrams of fast learning. Front. Cell. Neurosci. 14, 575915 (2020). 33250712 10.3389/fncel.2020.575915 PMC7676431

[49] B. M. Morrison , Oligodendroglia have a fundamental role in metabolic support of axons and contribute to neurodegeneration. Nature 487, 443–448 (2012). 22801498 10.1038/nature11314 PMC3408792

[50] S. Camandola, M. P. Mattson, Brain metabolism in health, aging, and neurodegeneration. EMBO J. 36, 1474–1492 (2017). 28438892 10.15252/embj.201695810 PMC5452017

[51] S. C. Cunnane , Brain energy rescue: An emerging therapeutic concept for neurodegenerative disorders of ageing. Nat. Rev. Drug Discov. 19, 609–633 (2020). 32709961 10.1038/s41573-020-0072-x PMC7948516

[52] D. Pathak, A. Berthet, K. Nakamura, Energy failure: Does it contribute to neurodegeneration? Ann. Neurol. 74, 506–516 (2013). 24038413 10.1002/ana.24014 PMC4092015

[53] M. Tang, S. H. Park, D. C. De Vivo, U. R. Monani, Therapeutic strategies for glucose transporter 1 deficiency syndrome. Ann. Clin. Transl. Neurol. 6, 1923–1932 (2019). 31464092 10.1002/acn3.50881 PMC6764625

[54] H. Berry, ANLS_STDP. GitLab. https://gitlab.inria.fr/hberry/anls_stdp. Deposited 24 May 2021.

