# Supplementary material for: Lactate supply overtakes glucose when neural computational and cognitive loads scale up
Source: Proc Natl Acad Sci U S A. 2022 Nov 14;119(47):e2212004119. doi: 10.1073/pnas.2212004119 (PMC9704697; doi:10.1073/pnas.2212004119)
Supplement: Supplementary File [file pnas.2212004119.sapp.pdf]

PNAS

Supporting Information for

Lactate supply overtakes glucose when neural computational and cognitive loads scale up

Yulia DEMBITSKAYA, Charlotte PIETTE, Sylvie PEREZ, Hugues BERRY, Pierre J MAGISTRETTI and Laurent VENANCE

Emails for correspondence:

laurent.venance@college-de-france.fr

pierre.magistretti@kaust.edu.sa

hugues.berry@inria.fr

This PDF file includes:

Supporting Information text:
Supplementary Materials and Methods
Supplementary Information for the mathematical model

Supporting Figures and Tables
Figures S1 to S7
Tables S1 to S4

SI references

Supporting Information Text

Supplementary Materials and Methods

Animals

Experiments were conducted in male Sprague Dawley rats (Charles River, L'Arbresle, France) $P_{30\text{-}35\ days}$ for brain slice patch-clamp and two-photon imaging, and $P_{7\text{-}9\ weeks}$ (weight: 250-300 g) for behavioral tasks and *in vivo* electrophysiology. C57BL/6 mice $P_{28\text{-}35}$ days were used for slice electrophysiology (*SI Appendix* Fig. S5). All experimental protocols were approved by the local animal welfare committee (Center for Interdisciplinary Research in Biology Ethics Committee) and EU guidelines (directive 2010/63/EU). Every effort was made to minimize animal suffering and to use the minimum number of animals per group and experiment. Animals were housed in standard 12-hour light/dark cycles and food and water were available *ad libitum*.

Patch-clamp whole-cell recordings in brain slices

Transverse hippocampal slices (350µm-thick) were prepared using a vibrating blade microtome (7000smz-2, Campden Instruments Ltd., UK) in ice-cold cutting solution containing (in mM): 93 N-Methyl-D-glucamine-Cl, 2.5 KCl, 30 $NaHCO_3$, 1.2 NaH_2PO4, 20 HEPES, 5 Na-ascorbate, 0.5 $CaCl_2$, 1 $MgSO_4$*7H_2O, 25 glucose, 3 Na pyruvate. The slices were transferred to the storage solution containing (in mM): 125 NaCl, 2.5 KCl, 5 glucose, 25 $NaHCO_3$, 1.25 NaH_2PO_4, 2 $CaCl_2$, 1 $MgCl_2$ with 10 µM pyruvic acid, for one hour at 34°C and then to room temperature. In a subset of experiments (Fig. 2D) storage solution containing 25 mM of glucose was used, as specified. All solutions were saturated with 95% O_2 and 5% CO_2. For whole-cell recordings from CA1 pyramidal neurons, borosilicate glass pipettes of 3-5 MΩ resistance were filled with (in mM): 127 K-gluconate, 30 KCl, 10 HEPES, 10 phosphocreatine (or 5 as specified in *SI Appendix* Fig. S4), 4 Mg-ATP (or 2 as specified in *SI Appendix* Fig. S4), 0.3 Na-GTP, 0.1 EGTA (adjusted to pH 7.35 with KOH). The composition of the extracellular solution was (mM): 125 NaCl, 2.5 KCl, 5 (or 25 as specified in Fig. 2D) glucose, 25 $NaHCO_3$, 1.25 NaH_2PO_4, 2 $CaCl_2$, 1 $MgCl_2$ and 10 µM pyruvic acid, through which 95% O_2 and 5% CO_2 was bubbled. Signals were amplified with EPC10-2 amplifiers (HEKA Elektronik, Lambrecht, Germany). Current- and voltage-clamp recordings were sampled at 20 kHz, with the Patchmaster v2x32 program (HEKA Elektronik). All recordings were performed at 35°C, using a temperature control system (Bath-controller V, Luigs & Neumann, Ratingen, Germany) and slices were continuously perfused at a rate of 2 ml/min.

Synaptic plasticity induction protocols

Synaptic responses in CA1 pyramidal cells were evoked by electrical stimulations of Schaeffer's collaterals with concentric bipolar electrodes (Phymep, Paris, France) placed in *stratum radiatum* area of hippocampus, at a distance exceeding 200 µm from the recording site. Electrical stimulations were monophasic, at constant current (ISO-Flex stimulator, AMPI, Jerusalem, Israel). Currents were adjusted to evoke 100-300 pA EPSCs. Repetitive control stimuli were applied at 0.1 Hz. Recordings were made over a period of 10 min at baseline, and for at least 60 min after the synaptic plasticity induction protocols; long-term changes of synaptic weight were measured in the last 10 min. Experiments were excluded if the mean input and series resistance (Ri and Rs, respectively) varied by more than 20% through the experiment.

Theta-burst stimulation (TBS). TBS of the Schaeffer's collaterals consisted of 10 bursts (single burst: 4 stimuli of 0.2 ms duration at 100 Hz) repeated at 5 Hz. TBS were applied either as a single TBS (1-TBS; Fig. 3A) or repeated 5 times at 0.1 Hz (5-TBS).

Spike-timing-dependent plasticity (STDP) protocols. STDP protocols consisted of pairings of pre- and postsynaptic stimulations separated by a fixed time interval (Δt_{STDP}=+10-15 ms, *i.e.* presynaptic stimulation preceded postsynaptic activation) (1,2). Presynaptic stimulations corresponded to Schaeffer's collaterals stimulations and the postsynaptic stimulation of an action potential (AP) evoked by a depolarizing current step (10 ms duration) in the recorded CA1 pyramidal cell. In a subset of experiments (Fig. 3C and 3D), 2 postsynaptic APs were elicited, as specified. Paired stimulations were repeated *n* times at a *f* frequency. In Figure 1 and *SI Appendix* Figure S1, we used STDP with 50 pairings at 0.5 Hz. In Figure 2 and 3, the number of postsynaptic APs (1 or 2), number of pairings (25, 50 or 100) and frequency (0.5 or 1 Hz) were varied.

Patch-clamp data analysis

Off-line analysis was performed with Fitmaster (Heka Elektronik), Igor-Pro 6.0.3 (Wavemetrics, Lake Oswego, OR, USA) and custom-made software in Python 3.0. Statistical analysis was performed with Prism 5.02 software (San Diego, CA, USA). We individually measured and averaged 60 successive EPSCs, comparing the last 10 min of the recording with the 10-min baseline recording in each condition using t-test. In all cases "n" refers to a single cell experiment from a single brain slice. All results are expressed as mean±SEM. Statistical significance was assessed by two-tailed student t-tests (unpaired or paired t-tests) or one-way ANOVA (with Newman-Keuls post hoc test) when appropriate, using the indicated significance threshold (*p*).

Two-photon imaging combined with whole-cell patch-clamp recordings

Morphological tracer Alexa Fluor 594 (50 µM) (Invitrogen, Waltham, MA, USA) and calcium-sensitive dye Fluo-4F (250 µM) (Invitrogen) were added to the intracellular solution. Cells were identified under Scientifica TriM-

Scope II system (LaVision, Germany), with a 60x/1.00 water-immersion objective. Alexa Fluor 594 and Fluo-4F were excited at 830 nm wavelength (femtoseconds IR laser Chameleon MRU-X1; Coherent, UK), and their fluorescence were detected with photomultipliers within 525/50 and 620/60 nm ranges, respectively. Line-scan imaging at 200 Hz was performed to obtain calcium signals in the dendritic shaft and spines and was synchronized with patch-clamp recordings. In each recording, we produced somatic depolarization and monitored maximal Ca2+ elevations to verify linear dependence of Fluo-4F Ca2+ signals (nonlinearity was below 20%) and that Ca2+ transients were below saturation level. The changes in baseline Ca2+ level were monitored as the ratio between the baseline Fluo-4F and Alexa Fluor 594 fluorescence. The cell was discarded for ratio>20%. The dark noise of the photomultipliers was collected when the laser shutter was closed in every recording. One or two bAPs evoked by a depolarizing current step (10 ms) were applied to monitor dendritic calcium transients. Electrophysiological data and calcium transients were analyzed with Fitmaster (Heka Elektronik) and custom-made software in Python 3.0 (https://github.com/calciumfilow/calcium-trace-extractor). The measurements of calcium transient were represented as $\Delta G/R$: $(G_{peak}-G_{baseline})/(R_{baseline}-R_{dark\ noise})$. Baseline Ca2+ signals were represented by baseline G/R, $(G_{baseline}-G_{dark\ noise})/(R_{baseline}-R_{dark\ noise})$, where G is the Fluo-4F fluorescence, and R is Alexa Fluor 594 fluorescence. $G_{baseline}$, $R_{baseline}$ and G_{peak} were obtained from the parameters of the bi-exponential fitting model in each trial and then averaged between 5-6 repetitions for each condition; the bi-exponential fitting was chosen to maximize the fit of the calcium-evoked events and gave better fit quality than single- and triple-exponential fitting. $G_{dark\ noise}$ and $R_{dark\ noise}$ are the dark currents of the photomultipliers. We ensured that $R_{baseline}$ and $G_{baseline}/R_{baseline}$ ratio did not exceed 20% over recording. The statistical significance was tested using a paired or unpaired Student's t-test.

Chemicals

1,4-Dideoxy-1,4-imino-D-arabinitol hydrochloride (DAB) (10µM) and DAB+L-lactate (10mM) were dissolved directly in the extracellular solution. (+)-5-methyl-10,11-dihydroxy-5H-dibenzo(a,d)cyclohepten-5,10-imine (MK801) (1mM), Na-oxamate (6mM), Na-oxamate (6mM) + L-pyruvate (10mM), Na-oxamate (6mM) + K_2NADH (4mM), D-Mannoheptulose (10µM), D-Mannoheptulose (10µM) + Na-oxamate (6mM), were added to intracellular recording solution. Na-oxamate (50mM) was dissolved directly in the saline solution for *in vivo* experiments. All chemicals were purchased from Sigma (Saint-Quentin Fallavier, France) except for MK801 (Tocris, Ellisville, MO, USA). For patch-clamp experiments, drugs were applied intracellularly (noted: i-drugs), ensuring specific intracellular effect in the sole recorded neuron without affecting the neighboring neurons or astrocytes; in few cases (Fig. 1E), drugs were applied extracellularly (noted: e-drugs).

Cannulas implants and *in vivo* microinjections

Under anesthesia with pentobarbital sodium (50 mg/kg i.p.), rats (250–300 g) were placed in a stereotactic apparatus and two holes were drilled to aseptically implant double guide cannulas (26GA, Bilaney, Germany) into dorsal hippocampi bilaterally (stereotaxic coordinates: anteroposterior =-4.0mm from bregma; mediolateral =-2.5mm from midline) (Fig. 4H), secured with dental cement. The two internal cannulas (33GA, Bilaney, Germany) were then inserted within the guide cannulas (dorsoventral =-2.2mm from bone surface). After surgery, 1mg/g i.p. of analgesic agent (Metacam, 1.5mg/ml solution) was administered for 3 days. Rats were allowed to recover from surgery for 7 days before behavioral test beginning. Rats were injected bilaterally through the stainless-steel cannulas 45 min before the familiarization phase of behavioral testing. Cannulas were briefly connected to 10 µL Hamilton syringes by means of polyethylene tubes. Rats were injected with either saline (0.8 mM sterile NaCl) or with Na-oxamate (50 mM) diluted in saline; 1-2 µl per hippocampus using automatic infusion pump at a speed of 0.2 µl/min.

Procedures for novel object recognition (NOR) and object-in-place (OiP) tasks

NOR and OiP tasks were run in a black Plexiglas training arena (100×100cm with 50 cm walls) in a sound-attenuated room with a controlled light intensity of 50 lx. The objects used were made out of plastic (laboratory cylinders and Lego blocks) and were selected to induce comparable attraction (*SI Appendix* Fig. S7A-B). The objects were fixed on the arena floor with a 10 cm distance from the walls. The NOR (2 objects: A and B) and OiP (4 objects: A, B, C and D) tasks involved three sessions upon three consecutive days: habituation (day 1), familiarization (day 2) and test (day 3).

Habituation (NOR and OiP). On day 1, rats were introduced into the empty arena (in its center) for 10 min; this was repeated twice the first day with 4 h intersession interval.

NOR task. For the familiarization session on day 2, the rat was placed in the center of the arena and exposed to two identical objects for 10 min: two objects A for half of the animals and two objects B for another half of the rats. Then, rats were returned to their home cage. For the test session on day 3, the rat was placed back into the center of the arena for object discrimination and were exposed to one familiar object and a novel test object, B for group precedingly exposed to A-A and A for group exposed to B-B, for 10 min.

OiP task. For the familiarization (day 2), the rat was placed in the center of the arena and exposed to four different objects: A, B, C, D, located clockwise as A-B-C-D, for 10 min. For the test session on day 3, the rat

was placed back into the arena for object discrimination and were exposed to the exchanged position of two of the presented objects, C-B-A-D for half of the animals and A-D-C-B for another half, for 10 min.

NOR and OiP performance analysis. Rats were videotaped during familiarization and test sessions. Videos were analyzed and time spent on exploration each object (sniffing or licking) was measured during familiarization and test sessions. For assessing NOR and OiP memory performance, the calculation of object discrimination, the exploration time of the novel object was expressed as percentage of the total exploration time of familiar and novel objects during familiarization and test sessions. Then, we calculated the relative time of exploration per object and the preference index (%) in control (saline-injected rats) and in oxamate (Na-oxamate-injected rats).

In vivo electrophysiology in behaving rats during NOR and OiP task

e-fEPSP were measured in the left CA1 over the 3-day behavioral assessment in rats subjected to NOR (familiarization: A-A and test: A-B) or OiP (familiarization: A-B-C-D and test: C-B-A-D) tasks. A recording wire (stainless steel, 0.005" diameter) was stereotaxically implanted under pentobarbital-ketamine anesthesia (pentobarbital: 30 mg/kg i.p., Ceva Santé Animale, Libourne, France; ketamine: 27.5 mg/kg, i.m., Imalgène, Mérial, Lyon, France) in CA1 stratum radiatum along the guide cannula (anteroposterior: -4.1mm, mediolateral: 2.5, dorsoventral : 3.3), and a bipolar stimulating electrode (2 twisted wires, same as the recording electrode) in the ipsilateral Schaffer collaterals (anteroposterior: -4.4mm, mediolateral: 4.4, dorsoventral : 3.4). The rat body temperature was maintained using at 37°C during surgery. Rats were allowed at least a week of recovery before electrophysiological recordings and behavioral testing. Recordings were performed during 10-15 min in a 24x44cm Plexiglas box, 60 min before familiarization (before the oxamate or saline injection) and, 2 and 24 hours after familiarization. Schaffer e-fEPSPs were amplified, and acquired at 20 kHz using a KJE-1001 system (Amplipex, Szeged, Hungary). Test pulses (150-200 µs, 20-450 µA) were evoked every 30 seconds using a square pulse stimulator and stimulus isolator (Model 2100, AM-Systems, Sequim, WA, USA). Responses were analyzed offline using custom Matlab codes (2019b, The Mathworks, Natick, MA, USA) scripts. Traces was smoothed (linear averaging) over 0.15, 0.25 or 0.5 ms depending on the noise level. Detection of local extrema was performed to define the start and peak of the response, and to measure the e-fEPSP amplitude. Statistical analyses (two-tailed Student t-test) and calculation of plasticity ratio were made on 25 trials. The average baseline of e-fEPSP amplitude obtained at t=-60 min was used to normalize every individual e-fEPSP amplitude at +2 and +24 hours, which were then averaged to obtain the plasticity ratio. The F-statistic and *p*-value associated with the average vectors were obtained from MANCOVA test (two dependent variables: preference index and plasticity ratio; one factor: belonging to the saline- or oxamate-injected rat group). Pearson's correlation coefficients were computed for paired plasticity ratios measured at 2 or 24 hours after familiarization phase.

Histology

After the completion of NOR and OiP experiments, cannulas and recording and stimulation electrodes positions were examined. Rats were anaesthetized (sodium pentobarbital, 150mg/kg i.p.) and transcardially perfused with saline followed by 4% ice-cold paraformaldehyde in 0.1 M phosphate buffer. Brains were extracted, post-fixed in PFA (4% in PBS) for 2 days at 4°C and then cryoprotected in 30% sucrose solution for one week. Brains were cut in coronal 50 µm sections using a cryotome (HM400, Microm Microtech, Francheville, France) and slices were maintained in 0.1 M potassium-PBS (pH=7.4). The sections were Nissl-stained with thionin (Neurotrace 500/525, Thermofischer, Waltham, MA, USA), and images were acquired using a stereozoom microscope (Axiozoom, Zeiss, Oberkochen, Germany) and processed in ImageJ. Only the rats with cannula tips located bilaterally within the dorsal hippocampi were included in the data analysis.

Mathematical model

In the mathematical model, though, parameter calibration imposes to specify which cell type one considers. The vast majority of published models and data on the subject is specific of astrocytes; we opted for astrocytes as glial cells in the model. Our model therefore simulates the network of signaling and metabolic reactions occurring in a postsynaptic neuronal terminal and an interacting astrocyte shown in Figure 2A.

Stimulations. The membrane potential of the postsynaptic compartment V_n is given by:

$$C_m \frac{\mathrm{d}V_n}{\mathrm{d}t} = -g_L(V_n - E_L) - I_{NMDA} - I_{AMPA} - I_{CaL} + I_{\text{step}} \quad (1)$$

$$\frac{\mathrm{d}I_{\text{step}}}{\mathrm{d}t} = -\frac{I_{\text{step}}}{\tau_{\text{step}}} + AT \cdot DP_{\text{max}} \sum_i 1_{[t_D^i, t_D^i + DP_{\text{dur}}]}(t) \quad (2)$$

with the indicator function $1_{[a,b]}(x) = 1 \text{ if } x \in [a,b], 0 \text{ otherwise}$, t_D^i is the time of the beginning of ith depolarization of the postsynaptic neuron, DP_{max} is the amplitude of the depolarization current in the postsynaptic neuron and DP_{dur} its duration. τ_{step} is the time step of the step current, AT is the attenuation of the bAP at the postsynaptic compartment and the models for the ionic currents $I_{NMDA}, I_{AMPA}, I_{CaL}$ (Fig. 2A) are given in the Supplementary Information.

STDP 1bAP: For STDP protocols with a single bAP per postsynaptic stimulation, we emulated the bAP by incrementing the postsynaptic potential by a constant value, *i.e.*:

$$V_n\left({t_{bAP1}^i}^+\right) = V_n\left({t_{bAP1}^i}^-\right) + AT \cdot AP_{\text{amp}} \quad (3)$$

where $t_{bAP1}^i = t_D^i + \delta_1$is the time of the (first) bAP, peaking with a delay δ_1 after the depolarization onset and AP_{amp} is the amplitude of the bAP measured in the soma.

STDP 2bAP: For STDP protocols with two bAPs per postsynaptic stimulation, we added an additional bAP at time $t_{bAP2}^i = t_D^i + \delta_1 + \delta_2$ where δ_2 is the delay between the 2 bAPs:

$$V_n\left({t_{bAP2}^i}^+\right) = V_n\left({t_{bAP2}^i}^-\right) + \alpha AT \cdot AP_{\text{amp}} \quad (4)$$

where α is the attenuation of the second bAP compared to the first. During STDP, we first set the times of each presynaptic stimulation $t_{\text{pre}}^i = t_{\text{pre}}^1 + (i-1)/f$ where t_{pre}^1 is the time of the first presynaptic stimulation (arbitrary) and *f* is the stimulation frequency in Hz. The postsynaptic times are then set according to the spike timing Δt_{STDP}: $t_{bAP1}^i = t_{\text{pre}}^i + \Delta t_{\text{STDP}}$ and $t_D^i = t_{\text{pre}}^i + \Delta t_{\text{STDP}} - \delta_1$

TBS: one presynaptic theta burst is composed of 4 presynaptic stimulations at 100 Hz repeated 10 times at 5 Hz and the frequency of the theta-burst themselves is 0.1 Hz, *i.e.*:

$$t_{\text{pre}}^i = t_{\text{pre}}^1 + \frac{1}{100}\text{mod}(k,4) + \frac{1}{5}\left\lfloor\frac{k}{4}\right\rfloor + \frac{1}{0.1}\left\lfloor\frac{i-1}{40}\right\rfloor$$

where $\lfloor x \rfloor$ denotes the integer part of x, $\text{mod}(x,j) = x - j\lfloor x/j \rfloor$ is its modulo *j* and $k = \text{mod}(i-1,40)$. On the postsynapse, experiments show that during a presynaptic TBS, the probability for a presynaptic spike to trigger a postsynaptic bAP is circa 0.5, therefore we set $DP_{\text{max}} = 0$ (no postsynaptic current injected in TBS) and set AP_{max}= 0 for odd is.

STDP. The variation of postsynaptic cytosolic calcium concentration is computed as:

$$\tau_{Ca}\frac{\mathrm{d}Ca_n}{\mathrm{d}t} = -Ca_n - \frac{S_m v_n}{F} I_{NMDA} - \frac{S_m v_n}{F} I_{CaL} \quad (5)$$

where $S_m v_n$is the surface-to-volume ratio of the postsynaptic compartment and F the Faraday constant. In the model, both the cytosolic calcium and ATP concentrations drive a bistable internal signaling state summarized by the state variable $\rho \in [0,1]$ (3):

$$\tau_\rho \frac{\mathrm{d}\rho}{\mathrm{d}t} = -\rho(1-\rho)(\rho^* - \rho) - LTD_{\text{max}}\rho \mathbf{1}_{[LTD_{\text{start}},+\infty[}(Ca_n) + LTP_{\text{max}}(1-\rho)\mathbf{1}_{[LTP_{\text{start}},+\infty[}(Ca_n) - M_{\text{max}}\rho\, \mathbf{1}_{[0,ATP_{\text{Thr}}]}(ATP_n) \quad (6)$$

where ATP_n is the concentration of ATP in the postsynaptic compartment, LTD_{max}, LTP_{max} and M_{max} are, respectively, the amplitudes of the calcium-gated LTD and LTP and of the ATP-gated depotentiation and LTD_{start}, LTP_{start} and ATP_{Thr} their respective thresholds. ρ^* sets the value of the unstable (intermediate) steady-state of the bistable and τ_ρ the time scale to reach the two stables steady-states $\rho = 0$ and $\rho = 1$. Finally, the synaptic weight is taken an affine function of the signaling state variable: $w(t) = 1 + \beta_\rho \rho(t)$ (7)

Astrocyte-neuron lactate shuttle. To derive the time course of ATP_n in eq. (6) as a function of the pre- and post-synaptic stimulations, we used the model developed by Jolivet *et al* (2015) (4). In both the postsynaptic terminal and a nearby interacting astrocyte (Fig. 2A), the model accounts for glycolysis, *i.e.* the production of pyruvate from glucose with glyceraldehyde-3 phosphate (GAP) and phosphoenolpyruvate (PEP) as intermediates as well as pyruvate formation from lactate by lactate dehydrogenase (LDH). The model also incorporates the production of NADH from pyruvate by the TCA cycle in the mitochondria, resulting ATP production by the electron transport chain as well as NADH shuttling from the cytosol to the mitochondria. Glucose and lactate are exchanged between the cytosol of the two compartments and the periplasmic extracellular medium via transporters, including MCT2 for lactate transport from the periplasmic volume to the postsynaptic compartment. Diffusive transport also occurs between the periplasmic volume and reservoir solutions with fixed concentrations (bath solution or blood capillaries of the slices). In addition, the model accounts for the activity-dependent dynamics of sodium ions in both compartments. Presynaptic stimulations trigger Na influx via EEAT2 channels in the astrocyte and voltage-gated sodium channels (VGSC) and (partly) AMPA receptors in the postsynaptic compartment. Sodium is then pumped back by Na,K-ATPases (ATPase), consuming ATP in the process.

We have implemented the model of Jolivet *et al* (2015) (4) *in extenso*. Our only change has been to substitute the Hodgkin-Huxley equation used for the postsynaptic membrane voltage in (30) by our eq. (1). This made us slightly adapt the maximum conductance for the postsynaptic VGSC, the strength of the effect of the presynaptic stimulation on the astrocyte and the leak conductance. All the remaining of the model, *i.e.* the 28 ODEs and the 80+ remaining parameters were taken unchanged from (4).

Parameter estimation. Coupling the two models (STDP + astrocyte-neuron lactate shuttle) left us with 27 parameters to estimate (*SI Appendix* Table S2). We used a subset of our experimental data (training set), whereas validation was carried out by checking the accuracy of the model output for experimental conditions that were not used in parameter estimation (pharmacology perturbation experiments, changes of extracellular glucose concentrations, different stimulation protocols). See Supplementary Information for a complete description of the model as well as parameter estimation strategy and values.

Code availability: Computer code for the model is publicly available at https://gitlab.inria.fr/hberry/anls_stdp

Supplementary Information Materials and Methods

Mathematical model

Ionic currents of the postsynaptic compartment membrane. The AMPA current was modeled as a double exponential conductance (5):

$$I_{AMPA} = \left[g_{AMPA\text{max}} \sum_i \left(\exp\left(-\frac{t-t^i_{\text{pre}}}{\tau_{AMPA1}}\right) - \exp\left(-\frac{t-t^i_{\text{pre}}}{\tau_{AMPA2}}\right)\right) \mathbf{1}_{[t^i_{\text{pre}},+\infty[}(t)\right](V_n - E_{AMPA}) \quad \text{(S1)}$$

where t^i_{pre} is the time of the ith presynaptic stimulation, $g_{AMPA\text{max}}$ the maximal AMPA conductance and τ_{AMPA1} and τ_{AMPA2} the two-time scales. The NMDAR was also modelled with a double exponential (6):

$$I_{NMDA} = \left[g_{NMDA\text{max}}\, B \sum_i \left(\exp\left(-\frac{t-t^i_{\text{pre}}}{\tau_{NMDA1}}\right) - \exp\left(-\frac{t-t^i_{\text{pre}}}{\tau_{NMDA2}}\right)\right) \mathbf{1}_{[t^i_{\text{pre}},+\infty[}(t)\right](V_n - E_{NMDA}) \quad \text{(S2)}$$

where the magnesium block $B = \left(1 + [\text{Mg}^{2+}]/3.57\exp(-0.062V_n)\right)^{-1}$. The VGCC current was modelled after (7) as a high-voltage L-type current:

$$I_{CaL} = g_{CaL\text{max}}\, s^2 u (V_n - E_{Ca}) \quad \text{(S3)}$$

with activation and inactivation functions given by:

$$\frac{\text{d}s}{\text{d}t} = 50(1-s)\exp\left(\frac{V_n+29.06}{15.9}\right) - 80s\exp\left(\frac{-V_n-18.66}{25.6}\right) \quad \text{(S4)}$$

and

$$\frac{\text{d}u}{\text{d}t} = (1-u)\exp\left(\frac{-V_n-48}{18.2}\right) - u\exp\left(\frac{V_n+48}{83}\right) \quad \text{(S5)}$$

The voltage-gated sodium current was taken from (4): $I_{Na} = g_{Na\text{max}}\, {m_\infty}^3 h(V_n - E_{Nan})$ (S6)

where the steady-state activation was $m_\infty = \alpha_m/(\alpha_m + \beta_m)$ with $\alpha_m = -0.1\,(V_n + 33)/[\exp(-0.1(V_n+33)) - 1]$ and $\beta_m = 4\exp(-(V_n + 58)/12)$ and the inactivation was given by:

$$\frac{\text{d}h}{\text{d}t} = 0.07(1-h)\exp\left(\frac{-V_n-50}{10}\right) - \frac{h}{\exp\left(\frac{-V_n-20}{10}\right)+1} \quad \text{(S7)}$$

Note that I_{Na} does not contribute to the evolution of the membrane potential in eq.(1) since eq.(1) does not include the spike currents but emulates the bAP using a simple increment of the potential at the spiking time followed by an exponential decay.

Astrocyte-neuron lactate shuttle. We implemented the model of Jolivet *et al* (2015) (4) where S_x denotes the concentration of chemical species S in compartment $x = \{a, n\}$ for astrocyte or postsynaptic neuronal, respectively. We give below a complete depiction of the equations and parameters of the model, but interested readers should refer to the original paper for further specifics.

The cytosolic sodium concentration is given by

$$\frac{\text{d}Na_x}{\text{d}t} = \frac{S_m v_x}{F} g_{Nax}(E_{Nax} - V_x) - 3S_m v_x k_{\text{pump}x} Na_x \frac{ATP_x}{1 + ATP_x/K_{\text{Mpump}}} + J_{\text{stim}x} \quad \text{(S8)}$$

with the Nernst potential $E_{Nax} = RT/F\,\log(Na_e/Na_x)$, g_{Nax} the leak conductance and $k_{\text{pump}x}$ the maximal rate of the Na-K-ATPase pump in compartment *x*. $J_{\text{stim}x}$ is the sum of sodium influxes triggered by the stimulations. In the postsynaptic compartment: $J_{\text{stim}n} = \frac{S_m v_x}{F}\left(-\frac{2}{3}I_{AMPA} - I_{Na}\right)$ (S9)

while in the astrocyte: $J_{\text{stim}a} = 2.25 \times 10^{-5} \times 1500 f \mathbf{1}_{[t^1_{\text{pre}}, t^N_{\text{pre}}]}(t)$ (S10)

where N is the total number of presynaptic stimulations and f their frequency (4).
Cytosolic glucose concentrations are given by

$$\frac{\mathrm{d}GLC_n}{\mathrm{d}t} = Tg_{en}\left(\frac{GLC_e}{GLC_e+K_{tg}} - \frac{GLC_n}{GLC_n+K_{tg}}\right) - k_{HKPFKn}\frac{ATP_n}{1+\left(ATP_n/K_{I,ATP}\right)^4}\frac{GLC_n}{GLC_n+K_g} \quad \text{(S11)}$$

and

$$\frac{\mathrm{d}GLC_a}{\mathrm{d}t} = Tg_{ca}\left(\frac{GLC_c}{GLC_c+K_{tg}} - \frac{GLC_a}{GLC_a+K_{tg}}\right) + Tg_{ea}\left(\frac{GLC_e}{GLC_e+K_{tg}} - \frac{GLC_a}{GLC_a+K_{tg}}\right) - k_{HKPFKa}\frac{ATP_a}{1+\left(ATP_a/K_{I,ATP}\right)^4}\frac{GLC_a}{GLC_a+K_g} \quad \text{(S12)}$$

where GLC_e is the extracellular concentration of glucose, i.e. in the pericellular volume, while GLC_c is its – constant - concentration in the large (reservoir) volume of the bath solution and/or the blood vessels. Tg_{xy} is the constant of glucose transport between compartments x and y, k_{HKPFKx} is the maximal rate for the part of glycolysis from hexokinase to phosphofructokinase (lumped as a single equivalent reaction).

Glyceraldehyde-3 phosphate (GAP) concentrations are obtained with:

$$\frac{\mathrm{d}GAP_x}{\mathrm{d}t} = 2k_{HKPFKx}\frac{ATP_x}{1+\left(ATP_x/K_{I,ATP}\right)^4}\frac{GLC_x}{GLC_x+K_g} - k_{PGKx}GAP_x ADP_x \frac{N-NADH_x^c}{NADH_x^c} \quad \text{(S13)}$$

where k_{PGKx} is the maximal rate for the part of glycolysis from GAP dehydrogenase to enolase, $NADH_x^c$ is the concentration of NADH in the cytosol of compartment x and N is the total concentration of NADH, i.e. the sum of NADH and NAD+ concentration in the cytosol. Phosphoenolpyruvate (PEP) concentration obeys:

$$\frac{\mathrm{d}PEP_x}{\mathrm{d}t} = k_{PGKx}GAP_x ADP_x \frac{N-NADH_x^c}{NADH_x^c} - k_{PKx}PEP_x ADP_x \quad \text{(S14)}$$

with k_{PKx} the pyruvate kinase rate in x. Next, the dynamics of pyruvate concentration (PYR) is given by:

$$\frac{\mathrm{d}PYR_x}{\mathrm{d}t} = k_{PKx}PEP_x ADP_x - k_{LDH\text{on}x}PYR_x NADH_x^c + k_{LDH\text{off}x}LAC_x\left(N - NADH_x^c\right) - v_{\text{mitoin}x}\frac{PYR_x}{K_{Mmito}+PYR_x}\frac{N-NADH_x^m}{N-NADH_x^m+K_{MNADx}} \quad \text{(S15)}$$

Here $k_{LDH\text{on}x}$ and $k_{LDH\text{off}x}$ are the forward and reverse rates, respectively, of lactate dehydrogenase (LDH) in compartment x, $NADH_x^m$ is the concentration of NADH in the mitochondrion of x and $v_{\text{mitoin}x}$ is the maximal rate of the TCA cycle. Lactate (LAC) dynamics is obtained via:

$$\frac{\mathrm{d}LAC_n}{\mathrm{d}t} = k_{LDH\text{onn}}PYR_n NADH_n^c - k_{LDH\text{offn}}LAC_n\left(N - NADH_n^c\right) - Tl_{ne}\left(\frac{LAC_n}{LAC_n+K_{tlne}} - \frac{LAC_e}{LAC_e+K_{tlne}}\right) \quad \text{(S16)}$$

where Tl_{xy} is the constant of lactate transport between compartments x and y and LAC_e is the extracellular concentration of lactate, *i.e.* in the pericellular volume. Likewise, in the astrocyte:

$$\frac{\mathrm{d}LAC_a}{\mathrm{d}t} = k_{LDH\text{ona}}PYR_a NADH_a^c - k_{LDH\text{offa}}LAC_a\left(N - NADH_a^c\right) - Tl_{ae}\left(\frac{LAC_a}{LAC_a+K_{tlae}} - \frac{LAC_e}{LAC_e+K_{tlae}}\right) - Tl_{ac}\left(\frac{LAC_a}{LAC_a+K_{tlac}} - \frac{LAC_c}{LAC_c+K_{tlac}}\right) \quad \text{(S17)}$$

where Tl_{xy} is the constant of lactate transport between compartments x and y and LAC_c is the extracellular concentration of lactate in the reservoir volume. Likewise, the concentration of lactose in the pericellular extracellular medium is given by:

$$\frac{\mathrm{d}LAC_e}{\mathrm{d}t} = \frac{Tl_{ae}}{r_{ea}}\left(\frac{LAC_a}{LAC_a+K_{tlae}} - \frac{LAC_e}{LAC_e+K_{tlae}}\right) + \frac{Tl_{ne}}{r_{en}}\left(\frac{LAC_n}{LAC_n+K_{tlne}} - \frac{LAC_e}{LAC_e+K_{tlne}}\right) - Tl_{ec}\left(\frac{LAC_e}{LAC_e+K_{tlec}} - \frac{LAC_c}{LAC_c+K_{tlec}}\right) \quad \text{(S18)}$$

and that of glucose:

$$\frac{\mathrm{d}GLC_e}{\mathrm{d}t} = -\frac{Tg_{ea}}{r_{ea}}\left(\frac{GLC_e}{GLC_e+K_{tg}} - \frac{GLC_a}{GLC_a+K_{tg}}\right) - \frac{Tg_{en}}{r_{en}}\left(\frac{GLC_e}{GLC_e+K_{tg}} - \frac{GLC_n}{GLC_n+K_{tg}}\right) + Tg_{ce}\left(\frac{GLC_c}{GLC_e+K_{tg}} - \frac{GLC_e}{GLC_e+K_{tg}}\right) \quad \text{(S19)}$$

with r_{xy} the ratio between the volume of compartment x and that of compartment y. The concentration of cytosolic NADH ($NADH_x^c$) is obtained by integration of:

$$(1-\xi)\frac{\mathrm{d}NADH_x^c}{\mathrm{d}t} = k_{PGKx}GAP_x ADP_x \frac{N-NADH_x^c}{NADH_x^c} - k_{LDH\text{on}x}PYR_x NADH_x^c + k_{LDH\text{off}x}LAC_x\left(N - NADH_x^c\right) - T_{NADHx}\frac{R^-{}_x}{R^-{}_x+M_{cytox}}\frac{R^+{}_x}{R^+{}_x+M_{mitox}} \quad \text{(S20)}$$

where T_{NADHx} is the maximal rate for NADH shuttling from cytosol to the mitochondria sub-compartment, ξ is the relative mitochondria volume, $R^{-}{}_{x} = \frac{NADH_x^c}{N-NADH_x^c}$ and $R^{+}{}_{x} = \frac{N-NADH_x^m}{NADH_x^m}$. For the mitochondrial concentration of NADH, one gets:

$$\xi \frac{\mathrm{d}NADH_x^m}{\mathrm{d}t} = T_{NADHx} \frac{R^{-}{}_{x}}{R^{-}{}_{x}+M_{cytox}} \frac{R^{+}{}_{x}}{R^{+}{}_{x}+M_{mitox}} + 4v_{\text{mitoin}x} \frac{PYR_x}{K_{Mmito}+PYR_x} \frac{N-NADH_x^m}{N-NADH_x^m+K_{MNADx}} - v_{\text{mitoout}x} \frac{O_{2_x}}{K_{O2mito}+O_{2_x}} \frac{ADP_x}{ADP_x+K_{MADPx}} \frac{NADH_x^m}{NADH_x^m+K_{MNADHx}} \quad \text{(S21)}$$

where $v_{\text{mitoout}x}$ is the maximal rate of the electron transport chain in *x* and O_{2_x} the oxygen concentration in this compartment. ATP concentration in the postsynaptic compartment is given by:

$$\left(1-\frac{dAMP_n}{dATP_n}\right)\frac{\mathrm{d}ATP_n}{\mathrm{d}t} = -2k_{HKPFKn}\frac{ATP_n}{1+\left(ATP_n/K_{I,ATP}\right)^4}\frac{GLC_n}{GLC_n+K_g} + k_{PGKn}GAP_nADP_n\frac{N-NADH_n^c}{NADH_n^c} + k_{PKn}PEP_nADP_n - J_{ATPasesn} - S_mV_nk_{\text{pump}n}Na_n\frac{ATP_n}{1+ATP_n/K_{\text{Mpump}}} + 3.6v_{\text{mitoout}n}\frac{O_{2n}}{K_{O2mito}+O_{2n}}\frac{ADP_n}{ADP_n+K_{MADPn}}\frac{NADH_n^m}{NADH_n^m+K_{MNADHn}} + k_{\text{CKon}n}PCr_nADP_n - k_{\text{CKoff}n}(C-PCr_n)ATP_n \quad \text{(S22)}$$

with $J_{ATPasesn}$ a parameter accounting for ATPase activities outside Na-K-ATPases, $k_{\text{CKon}n}$ and $k_{\text{CKoff}n}$ the forward and backward rates of creatine kinase, respectively, and *C* the total concentration of creatine plus phosphocreatine.

$dAMP_n/dATP_n$, the ratio between deoxyAMP and deoxyATP is computed with $dAMP_x/dATP_x = -1 + 0.5q_{AK} - 0.5\sqrt{u_x} + Aq_{AK}/\left(ATP_x\sqrt{u_x}\right)$ where q_{AK} is the adenylate kinase equilibrium constant, $A = AMP_x + ADP_x + ATP_x$ is the total adenine nucleotide concentration and $u_x = q_{AK}{}^2 + 4q_{AK}(A/ATP_x - 1)$. Similarly, the concentration of ADP is computed from that of ATP using $ADP_x = 0.5ATP_x\left(-q_{AK} + \sqrt{u_x}\right)$.

Now, in the astrocyte, the concentration of ATP is given by:

$$\left(1-\frac{dAMP_a}{dATP_a}\right)\frac{\mathrm{d}ATP_a}{\mathrm{d}t} = -2k_{HKPFKa}\frac{ATP_a}{1+\left(ATP_a/K_{I,ATP}\right)^4}\frac{GLC_a}{GLC_a+K_g} + k_{PGKa}GAP_aADP_a\frac{N-NADH_a^c}{NADH_a^c} + k_{PKa}PEP_aADP_a - J_{ATPasesa} - \frac{3}{4}J_{pumpa0} - \frac{7}{4}S_mV_ak_{\text{pump}a}Na_a\frac{ATP_a}{1+ATP_a/K_{\text{Mpump}}} + 3.6v_{\text{mitoout}a}\frac{O_{2a}}{K_{O2mito}+O_{2a}}\frac{ADP_a}{ADP_a+K_{MADPa}}\frac{NADH_a^m}{NADH_a^m+K_{MNADHa}} + k_{\text{CKon}a}PCr_aADP_a - k_{\text{CKoff}a}(C-PCr_a)ATP_a \quad \text{(S23)}$$

where phosphocreatine concentrations are given by

$$\frac{\mathrm{d}PCr_x}{\mathrm{d}t} = -k_{\text{CKon}x}PCr_xADP_x + k_{\text{CKoff}x}(C-PCr_x)ATP_x \quad \text{(S24)}$$

Finally, oxygen concentrations are obtained through:

$$\frac{\mathrm{d}0_{2x}}{\mathrm{d}t} = \frac{PS_{cap}}{v_x}\left(K_{O2}\left(\frac{Hb.OP}{0_{2c}}-1\right)^{-1/n_h} - 0_{2x}\right) - 0.6v_{\text{mitoout}x}\frac{O_{2x}}{K_{O2mito}+O_{2x}}\frac{ADP_x}{ADP_x+K_{MADPx}}\frac{NADH_x^m}{NADH_x^m+K_{MNADHx}} \quad \text{(S25)}$$

where PS_{cap}/v_x is the oxygen transport constant from the reservoir to the cells and 0_{2_c} oxygen concentration in the reservoir.

Numerical integration. The system of ODEs consisting of eq.(1)-(7) and eq.(S1)-(S25) was integrated using a variable-order adaptive-stepsize stiff solver (ode15s in Matlab®), interrupting integration at every stimulation events (times $t_D^i, t_D^i + DP_{\text{dur}}$, t_{bAP1}^i and t_{bAP2}^i if relevant) to account for the discontinuities of eq.(2-4). The system was first integrated for 2×10^5 seconds in the absence of any electrical stimulation to guaranty that the initial state (before electrical stimulation) is the stable steady-state. After electrical simulation (TBS or STDP), the system was integrated for 45 min. The state of the system 45 min after the stimulation sets the value of the synaptic weight (eq.7) after the stimulation, thus the potential expression of LTP.

Parameter estimation. To calibrate the model, we used the following strategy. All the parameters of equations (S3) to (S25) kept the values set by (4) (*SI Appendix* Table S2) except for $g_{Na\max}$ (eq. S6), g_{L}(eq.1) and the prefactor of eq.(S10), that were modified as explained below. Therefore, parameter estimation was almost completely restricted to the parameters of eq.(1-7). Together, this represented 27 parameters to estimate (*SI Appendix* Table S2). Parameter values were estimated on a subset of our available experimental data (see below) whereas validation was carried out by checking the accuracy of the model output for experimental conditions that were not used in parameter estimations.

1. The parameters of the postsynaptic stimulations DP_{max}, τ_{step}, AP_{amp} and α as well as the delays δ_1 and δ_2 and the leak conductance g_{L} were fitted to the experimental traces of the postsynaptic membrane potential as measured in the soma (*SI Appendix* Fig. S2B). The attenuation factor of the bAP between the soma and the postsynaptic compartment, AT, was set to a value that roughly corresponds to a synapse located at mid-distance between the soma and the dendrite end according to (8) (see their Fig 4D).

2. $g_{AMPA\text{max}}$, τ_{AMPA1} and τ_{AMPA2} were set to yield EPSPs of 2 mV amplitude, with short onset and a decay time scale around 10 ms, as measured in dendrites by (9). τ_{NMDA1} and τ_{NMDA2} were set to yield a NMDA-component for the calcium influx that rises fast and takes roughly 200 ms to get back to zero and $g_{NMDA\text{max}}$ was fixed so that each presynaptic spike increases the cytosolic calcium level by 0.17 mM at -70 mV (10). $g_{CaL\text{max}}$ was set so that one bAP (on top of the depolarizing current) triggers a calcium influx of 400 nM amplitude at the synapse in the absence of presynaptic stimulation (11). We also checked that with these parameter values, the amplitude of the calcium peak triggered by a postsynaptic stimulation comprising two bAPs is indeed roughly twice the amplitude obtained with a single bAP (*SI Appendix* Fig. S2B) as measured in our experimental setup both in spines and shafts (*SI Appendix* Fig. S6).

3. In the original model (30), the membrane voltage of the presynaptic compartment is modelled as a Hodgkin-Huxley equation with variable Nernst potentials for Na. Because of the variable Nernst potential, this model exhibits strong spike-frequency adaptation so the postsynaptic neuron quickly ceases to emit spikes after the onset of the stimulation. Since our membrane voltage for the postsynaptic compartment does not exhibit spike-frequency adaptation, we had to adapt a pair of parameters to guaranty that the electrical stimulations employed in the experiments used to calibrate the model by (4) (their Fig. 4A, with experimental data taken from (11) will still yield the correct time course in our model. We therefore adapted the values of $g_{Na\text{max}}$ and the prefactor of eq.(S10) so that the stimulation used in (11) (presynaptic stimulations for 20 sec) yielded in our model the same time-courses for $NADH_a^c$ and $NADH_n^m$ as their experimental measurements (their Fig. 4D). In particular (*SI Appendix* Fig. S2C) this stimulation leads to 1) a dip of $NADH_n^m$ of around -10%, peaking around the end of the effective stimulation, and converging back to baseline after 15-20 min, and 2) a delayed overshoot of $NADH_a^c$ that peaks at approx. +8% roughly 20 min after the end of the stimulation. Those dynamics reproduce previous experimental measurements (33), in both quantitative and qualitative terms.

4. The parameters related to the dynamics of the synaptic weight were set as follows. The calcium thresholds LTD_{start} and LTP_{start} were set so that a "standard" STDP protocol (1 bAP at 1 Hz) triggers LTP for positive spike timings that are not larger than 30 ms and for more than approx. 20 pairings (*SI Appendix* Fig. S2D). The values of LTD_{max}, LTP_{max}, ρ^* and β_ρ were fitted on the experimental measurements of the time course of the synaptic weight change for a STDP protocol with 1 bAP, 50 x at 0.5 Hz (Fig. 2B). Note that with 5-TBS, the experimental measurement of the final amplitude of the synaptic weight change was larger than with STDP (Fig. 2B), we thus adapted the value of β_ρ for 5-TBS (but kept the values of LTD_{max}, LTP_{max} and ρ^* to those obtained by fitting on STDP 0.5 Hz 50x).

5. Finally, we fixed the ATP threshold ATP_{Thr} to a value that allows discriminating between STDP 0.5 Hz 50 pairings (LTP) stimulations and 5-TBS with oxamate (no LTP). In particular, the astrocyte-neuron lactate shuttle model predicts that neuronal ATP, ATP_n, falls below 2 mM for 5-TBS in the presence of oxamate (Fig. 2C), but remains well above 2 mM for 5-TBS in control conditions and STDP 50 pairings at 0.5 Hz (in control and with oxamate). In the absence of experimental data to set the value of M_{max}, we used a value large enough to cancel LTP for 5-TBS in the presence of oxamate.

Simulation of pharmacological experiments. The action of pharmacological agents was emulated by changing the corresponding parameters to the following values:

1. *Oxamate*: the forward and backward rate constants of LDH in the postsynaptic neuronal compartment were divided tenfold, i.e. we set $k_{LDH\text{on}n} = 0.723$ mM^{-1}s^{-1} and $k_{LDH\text{off}n} = 0.0720$ mM^{-1}s^{-1}.
2. *Mannoheptulose:* the forward constant of the HKPFK reaction in the postsynaptic compartment was divided by 1000, *i.e.* we set $k_{HKPFKn} = 0.0504 \times 10^{-3}$ mM^{-1}s^{-1}
3. *Changes of glucose concentration in the bath:* was emulated by a corresponding change of the glucose concentration in the (constant) reservoir, GLC_c.
4. *Changes of NADH in the patch pipette:* were emulated by corresponding change of the total NADH+NAD$^+$ concentration *N* in the postsynaptic compartment (while *N* kept its control value of 0.212 mM in the astrocyte).

Supporting Figures and Tables

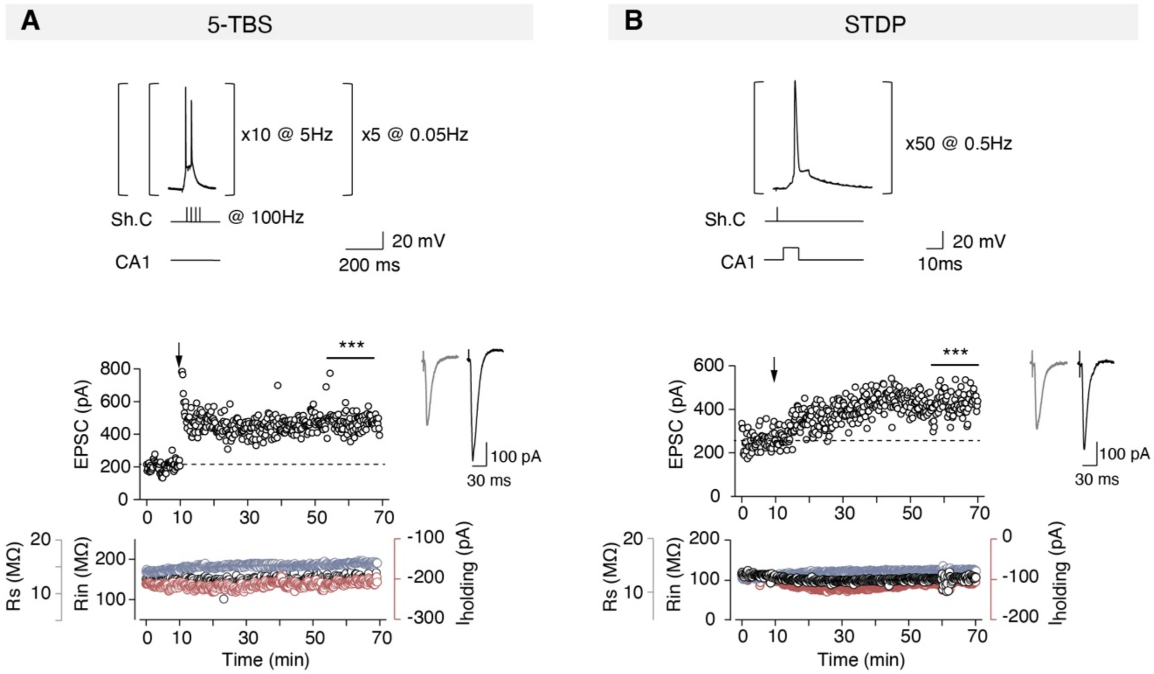

Fig. S1. Representative 5-TBS-LTP and STDP-LTP (50 pairings at 0.5 Hz) (related to the main Figure 1). (**A**) Example of LTP induced by 5-TBS (baseline: 205±4pA, increased by 127%, to 446±6pA, one hour after pairings.). Bottom, time course of Ri (baseline: 145±1MΩ and 50-60 min after pairings: 154±1MΩ; change of 6%), Rs (baseline: 14.14±0.03MΩ and 50-60 min after pairings: 16.31±0.02MΩ; change of 15%) and holding current $I_{holding}$ (baseline: -217±1pA and 50-60 min after pairings: -208±4pA; change of -4%). (**B**) Example of STDP-LTP induced by 50 pre-post pairings (spike timing=-8.7±0.4ms) (the mean baseline EPSC amplitude was 257±5pA before pairings and was increased by ~67% to 430±6pA one hour after pairings). Bottom, time course of Ri (baseline: 110±1MΩ and 50-60 min after pairings: 101±1MΩ; change of -8%), Rs (baseline: 12.72±0.02MΩ and 50-60 min after pairings: 14.05±0.03MΩ; change of 10%) and holding current $I_{holding}$ (baseline: -93±1pA and 50-60 min after pairings: -109±1pA; change of 18%).

Insets correspond to the average EPSC amplitude during baseline (grey traces) and the last 10 min of recording after STDP pairings (red traces). Statistics (student t-test, first vs last 10 min of recording): *** p<0.001.

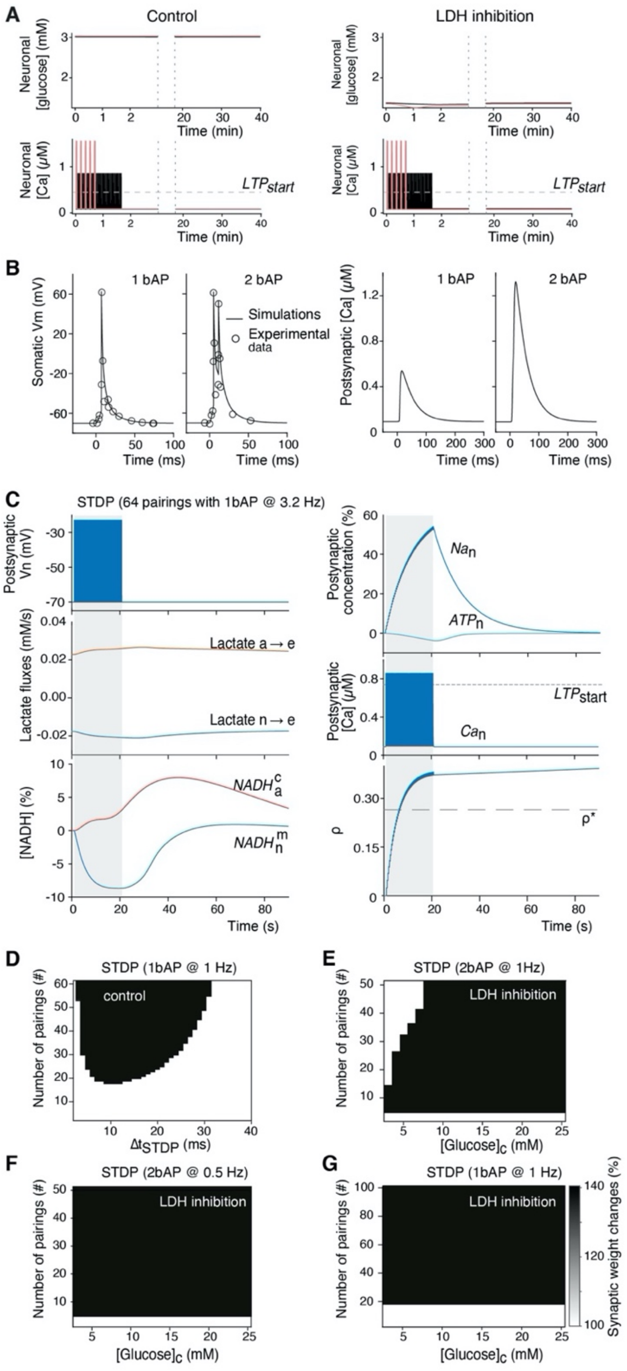

Fig. S2. Model calibration (related to the main Figure 2).

(A) Kinetics of neuronal glucose and calcium with 5-TBS (pink) or light STDP (50 pairings at 0.5Hz, black). (B) Experimental measurements (circles) and model prediction (line) of the somatic potential with 1 bAP or 2 bAP-stimulations and spike timing 10ms (*left*), together with the resulting calcium transients (*right*). (C) After calibration, the glia-neuron lactate shuttle part of the model reproduces experimental data in (*23*) where a 20 sec electrical stimulation of the neurons and astrocytes triggered a decay of neuronal mitochondrial NADH ($\boldsymbol{NADH_n^m}$) by 10% peaking around the end of the stimulation and converging back to baseline 15-20 min afterwards and a delayed overshoot of astrocytic cytosolic NADH ($\boldsymbol{NADH_a^c}$). (D) Prediction of synaptic plasticity by the model when the number of pairings and the spike timing vary in STDP (1 bAP at 1Hz) in control conditions. With the color code used, white means no plasticity, while the black zones correspond to LTP (E-G) explore the output of the model with LDH inhibited (oxamate), for STDP with 2 bAPs at 1Hz, 2 bAPs at 0.5Hz, and 1 bAP at 1 Hz, respectively, as a function of the number of pairings and the concentration of glucose in the bath solution.

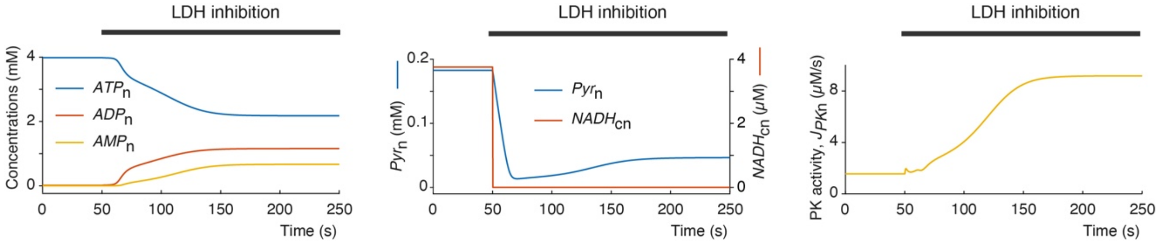

Fig. S3. Model prediction of the effects of LDH inhibition on neuronal ATP levels (related to Figure 2).
The model is initiated at time t=0 in control conditions. In control (t< 50s), ATP makes up roughly the totality of the 4 mM total adenosine phosphate concentration (*top*), the stationary level of pyruvate (Pyr_n) and cytosolic NADH ($NADH_{cn}$) in the neuron is large (*middle*), and neuronal glycolysis is low, as witnessed by the low activity of pyruvate kinase, PK (*bottom*). Oxamate addition in the neuron cytosol at t=50 sec rapidly switches the neuron to an oxidized redox state with a close to total depletion of cytosolic NADH. As a result, the ATP level drops well below 4 mM. Oxamate addition also results in neuronal glycolysis, as illustrated by the increase of pyruvate kinase activity. This restores significant levels of neuronal pyruvate and stabilizes ATP to roughly 50% of the total adenosine phosphate, *i.e.* slightly above 2 mM.

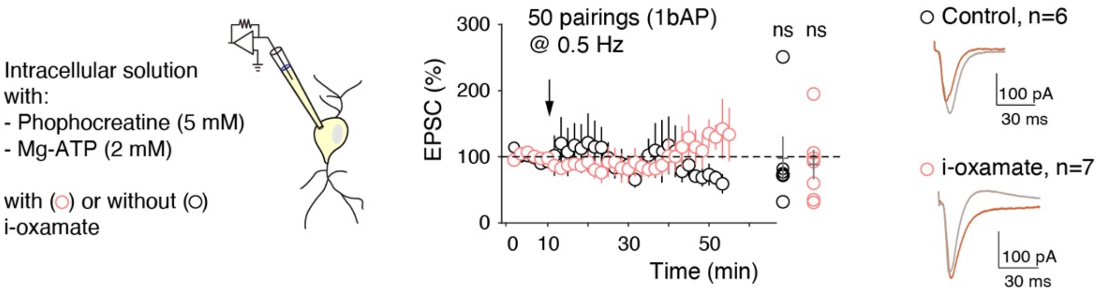

Fig. S4. STDP is prevented with low intracellular ATP (2 mM) and phosphocreatine (5 mM) (related to Figure 2).
Averaged time-course of the synaptic weight and EPSC amplitudes 45-55 min after STDP. When intracellular solution contained 2 mM of ATP and 5 mM phosphocreatine, LTP was not observed after STDP protocol in control or i-oxamate conditions. Only one significant LTP could be induced in control and i-oxamate conditions out of 6 and 7 cells, respectively. All data: mean±SEM. ns: not significant by two tailed t-test. See *SI Appendix* Table S3 for detailed data and statistics.

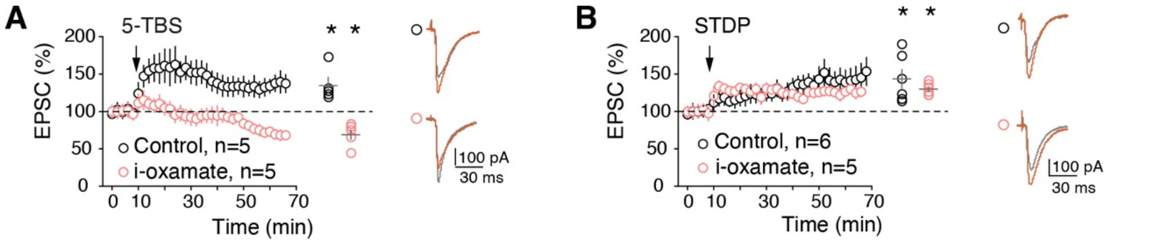

Fig. S5. LDH inhibition prevents 5-TBS-LTP and left unaffected STDP-LTP in adult mice.
Averaged time-course of the synaptic weight and EPSC amplitudes 50-60 min after 5-TBS or STDP in adult mice. Intracellular inhibition of LDH with i-oxamate show distinct effects on 5-TBS and STDP expression since 5-TBS did not induce plasticity whereas STDP triggered a potent LTP. Representative traces: 15 EPSCs averaged during baseline (grey) and 60 min (red) after protocols (arrows).All data: mean±SEM. *$p<0.05$; ns: not significant by two tailed t-test. See *SI Appendix* Table S3 for detailed data and statistics.

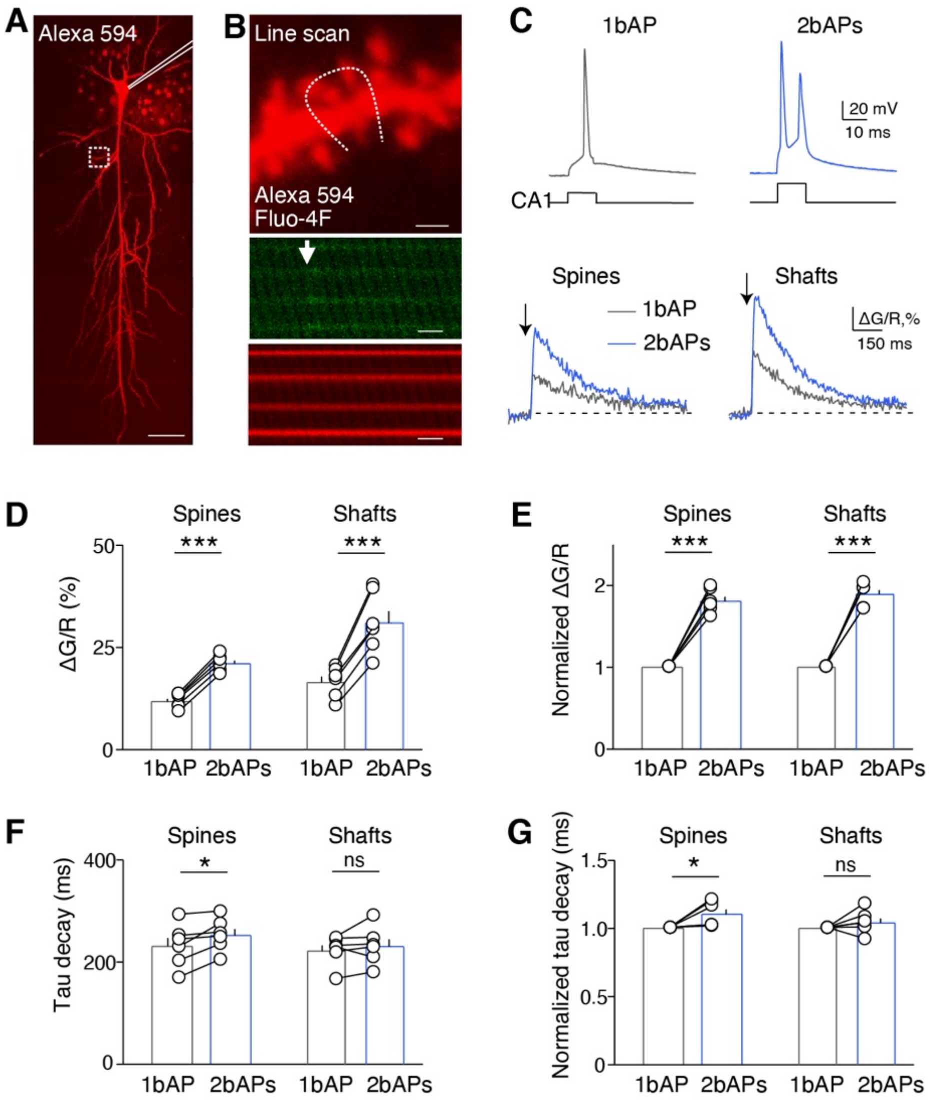

Fig. S6. Calcium transients in dendritic spines and shafts upon single or two bAPs.
(**A**) Combination of whole-cell recording of a CA1 pyramidal cell with two-photon imaging; the patch-clamp pipette is underlined in white and the dashed white square indicated the imaged dendritic area. The scanning areas had been selected on 50-150µm distance from soma. Scale bar: 30µm. (**B**) Line-scanning two-photon microscopy of Ca^{2+} transients in dendritic spines and adjacent shaft (top panel; scale bar: 2µm) filled with ratiometric indicators Fluo-4F (250µM; scale bar: 200ms) (middle panel) and Alexa Fluor 594 (50µM) (bottom panel; scale bar: 200ms). (**C**) A single or two bAPs were triggered in the recorded CA1 pyramidal cell by a postsynaptic current depolarization of 10 ms duration, and calcium transients were recorded in the dendritic spines and neighboring shafts. (**D** and **E**) Two bAPs induced larger increase of calcium in spines and shafts than a single bAP when evaluating ΔG/R (**D**) or the normalized ΔG/R (**E**). (**F** and **G**) Two bAPs induced a longer decay of the calcium transient in spines but not in shafts when compared to a single bAP, as estimated by the tau decay (**F**) and the normalized tau decay (**G**). Error bars represent the SEM. *: p<0.05; ***: p<0.001; ns: not significant.

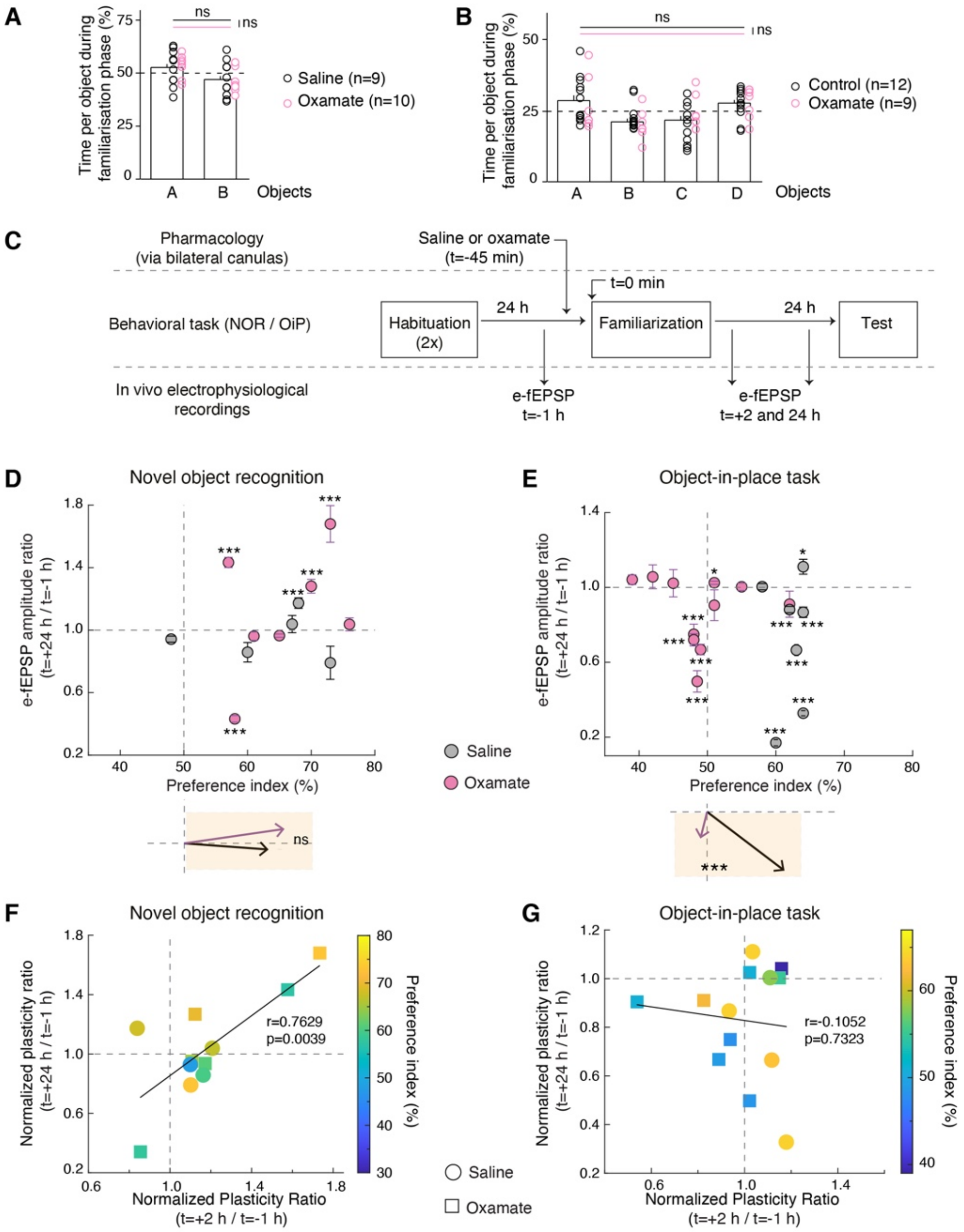

Fig. S7. ***In vivo* synaptic plasticity 24 hours after test for NOR and OiP tasks (related to Figure 4).** (**A**) Rats injected in CA1 with saline or oxamate solutions spent similar amount of time exploring A and B (saline: *p*=0.3750, oxamate: *p*=0.1661, saline *vs* oxamate: object A: *p*=0.5563, object B: *p*=0.5563, one-way ANOVA). (**B**) Saline- or oxamate-injected rats explored similarly A, B, C and D (saline: *p*=0.1342, oxamate: *p*=0.2178, saline vs oxamate: A: *p*=0.8826, B: *p*=0.1342, C: *p*=0.2566, D: *p*=0.6964, one-way ANOVA). A and B were common for both NOR and OiP tasks. (**C**) Behavioral experimental set-up for the NOR and OiP tasks and e-fEPSP recordings. (**D** and **E**) No difference for e-fEPSP amplitudes 24 hours after familiarization were observed between saline or oxamate-injected rats, with on average no plasticity for the NOR task (**D**) and a majority of LTD for the OiP task (**C**) (see average vectors in **D** and **E**). (**F** and **G**) Correlation between e-LFPs recorded 2 and 24 hours after familiarization in the NOR and OiP tasks, in relation with the preference index. NOR: significant positive correlation with the plasticity ratios decreasing from +2 to +24 hours, with larger LTPs remaining LTP at +24 hours, while smaller LTPs shifting into no plasticity or LTD (similar for both saline- and oxamate-injected rats). OiP: no correlation between plasticity at +2 and +24 hours with LTP at +2 hours turned either into no plasticity or LTD while the majority of LTD at +2 hours persisted in LTD at +24 hours. All data are presented as mean±SD. *: p<0.05; **: p<0.01; ***: p<0.001; by two tailed *t*-test.
See *SI Appendix* Tables S4C and S4D for detailed data and statistics.

Tables S1 (related to Figure 1):

	5-TBS			STDP (50 pairings @ 0.5 Hz)		
Experimental conditions	EPSC amplitude % of baseline (n)	t-test, *p* value	2-way ANOVA, *p* value	EPSC amplitude % of baseline (n)	t-test, p value	2-way ANOVA, *p* value
Control	152.4 ±13.8(14)	0.0056	<0.0001	138.3 ± 10.2 (8)	0.0070	<0.0001
i-MK801	89.7 ± 6.4 (7)	0.1562	0.0801	103.4 ± 7.0 (7)	0.6484	0.4800
DAB	89.6 ± 10.5 (6)	0.3691	0.0651	91.8 ± 9.5 (7)	0.4213	0.0838
DAB + e-lactate(1) (2)	142.6 ± 17.0(6)	0.0311	0.0032	159.3 ± 24.1(7)	0.0383	0.0002
i-oxamate	91.7 ± 9.9 (9)	0.4600	0.0643	134.8 ± 14.0 (7)	0.0398	0.0008
i-oxamate + i-pyruvate	104.4 ± 13.3 (7)	0.7548	0.5110	-	-	-
i-oxamate + i-NADH(3)	158.5 ± 16.0 (6)	0.0145	<0.0001	-	-	-

(1), (2) and (3): rescue experiments *vs* corresponding control (t-test, *p* value)
(1) 5-TBS: control *vs* e-DAB + e-lactate, *p*=0.6735
(2) STDP (1 Hz, 50 pairings): control *vs* e-DAB + e-lactate, *p*=0.4155
(3) 5-TBS: control *vs* i-oxamate + i-NADH, *p*=0.7995

Table S2. Parameter values of the mathematical model

Parameters	Description	Value	Unit
**Parameters estimated from our experimental data**			
g_L	Postsynaptic leak conductance	0.50	mS/cm^2
τ_{step}	Postsynaptic depolarization current time constant	15	ms
AT	Attenuation factor at the synapse	0.34	-
DP_{max}	Amplitude of the postsynaptic depolarization	1.50 (STDP 1bAP)<br>2.85 (STDP 2bAP)<br>0 (TBS)	mA/cm^2
DP_{dur}	Duration of the postsynaptic depolarization	10 (STDP)<br>0 (TBS)	ms
AP_{amp}	Amplitude of the bAP at the soma	120 (STDP 1bAP)<br>114 (STDP 2bAP)<br>130 (TBS)	mV
δ_1	Delay between depolarization onset and first bAP	7 (STDP 1bAP)<br>5.5 (STDP 2bAP)	ms
δ_2	Delay between first and second bAP	6.3 (STDP 2 bAP)	ms
α	Additional attenuation of the second bAP	0.65 (STDP 2 bAP)	-
τ_{Ca}	Time scale of cytosolic Ca dynamics	40	ms
τ_ρ	Time scale of the internal state variable	103.15	s
ρ^*	Unstable middle point of the bistable	0.257	-
LTD_{max}	Maximal amplitude of the LTD	70	-
LTD_{start}	Ca-threshold for the LTD	0.45	µM
LTP_{max}	Maximal amplitude of the LTP	120	-
LTP_{start}	Ca-threshold for the LTP	0.74	µM
M_{max}	Max. amplitude of the ATP-gated depotentiation	30	-
ATP_{Thr}	ATP-threshold of the ATP-gated depotentiation	1.87	mM
β_ρ	Final synaptic weight increase in LTP	0.405 (STDP 1baP)<br>0.405 (STDP 2baP)<br>0.550 (TBS)	-
τ_{AMPA1}	AMPA conductance time constant 1	9.6	ms
τ_{AMPA2}	AMPA conductance time constant 2	7.0	ms
$g_{AMPA\text{max}}$	Max. AMPA conductance density	0.13	mS/cm^2
τ_{NMDA1}	NMDA conductance time constant 1	60.0	ms
τ_{NMDA2}	NMDA conductance time constant 2	1.0	ms
$g_{NMDA\text{max}}$	Maximal NMDA conductance density	4.64×10^{-4}	mS/cm^2
$g_{CaL\text{max}}$	Maximal CaL conductance density	0.0849	mS/cm^2
$g_{Na\text{max}}$	Maximal conductance density of VGSCs	11.5	mS/cm^2
**Fixed parameters and constants**			
$[\text{Mg}^{2+}]$	Magnesium concentration	1.0	mM
GLC_c	Glucose concentration in the bath solution in control	5.0	mM
E_{Ca}	Nernst Potential for Ca^{2+}	54[1]	mV
E_{NMDA}	Reversal potential NMDA receptors	0	mV
E_L	Reversal potential leak current	-70	mV
R	Gas constant	8.3145	J/mol/K
T	Temperature	310	K
F	Faraday Constant	96 485.3	C/mol
**Fixed parameters** taken from (*19*) with no change[2]			
$S_m v_x$	Surface/vol. ratio of compartment $x = \{a, n\}$	2.5×10^4	cm^{-1}
E_{AMPA}	Reversal potential AMPA receptors	0	mV
C_m	Membrane Capacitance postsynaptic compartment	10^{-3}	mF/cm^2
Na_e	Sodium extracellular concentration	150	mM
g_{Nax}	Na leak conductance in the postsynaptic compartment	0.0136 (*n*)<br>0.0061 (*a*)	mS/cm^2
V_a	Membrane voltage of the astrocyte	-70	mV
$k_{\text{pump}x}$	Max. rate Na-K-ATPases	2.2×10^{-6} (*n*)<br>4.5×10^{-7} (*a*)	cm/mM/s

K_{Mpump}	Affinity constant for ATP of Na-K-ATPases	0.5	mM
K_{tg}	Affinity constant of glucose transporters	8	mM
Tg_{xy}	Constant of glucose transport between compartments *x* and *y*	0.0016 (*ca*)<br>0.0410 (*en*)<br>0.1470 (*ea*)<br>0.2390 (*ce*)	mM/s
k_{HKPFKx}	Maximal rate combined HK-PFK	0.0504 (*n*)<br>0.185 (*a*)	s^{-1}
K_g	Affinity for glucose HKPFK	0.05	mM
k_{PGKx}	Maximal rate PGK	3.97 (*n*)<br>135.2 (*a*)	$mM^{-1}s^{-1}$
N	Sum of NADH plus NAD^+ concentrations in control	0.212	mM
A	Total adenine nucleotide concentration	4.0 (*n*)<br>2.212 (*a*)	mM
C	Creatine plus phosphocreatine concentration	10	mM
q_{AK}	Adenylate kinase equilibrium constant	0.92	mM
k_{PKx}	Max. rate pyruvate kinase	36.7 (*n*)<br>401.7 (*a*)	$mM^{-1}s^{-1}$
$k_{LDH\text{on}x}$	Forward rate constant of LDH	72.3 (*n*)<br>1.59 (*a*)	$mM^{-1}s^{-1}$
$k_{LDH\text{off}x}$	Backward rate constant of LDH	0.720 (*n*)<br>0.071 (*a*)	$mM^{-1}s^{-1}$
LAC_c	Lactate concentration in the reservoir	0.55	mM
Tl_{xy}	Constant of lactate transport between compartments *x* and *y*	24.3 (*ne*)<br>106.1 (*ae*)<br>0.00243 (*ac*)<br>0.25 (*ec*)	mM/s
K_{tlxy}	Affinity constant of lactate transporters between *x* and *y*	0.74 (*ne*)<br>3.50 (*ae*)<br>1.00 (*ac*)<br>1.00 (*ec*)	mM
$v_{\text{mitoin}x}$	Maximal rate of the TCA cycle	0.1303 (*n*)<br>5.7 (*a*)	mM/s
K_{Mmito}	Affinity constant TCA cycle for pyruvate	0.04	mM
K_{MNADx}	Affinity constant TCA cycle for NAD	0.409 (*n*)<br>40.3 (*a*)	mM
$v_{\text{mitoout}x}$	Maximal rate of the electron transport chain (ETC)	0.164 (*n*)<br>0.064 (*a*)	mM/s
K_{O2mito}	Affinity constant of ETC for O_2	0.001	mM
K_{MADPx}	Affinity constant of ETC for ADP	3.410 (*n*)<br>0.483 (*a*)	μM
K_{MNADHx}	Affinity constant of ETC for NADH	44.4(*n*)<br>26.9 (*a*)	μM
T_{NADHx}	Maximal rate for NADH shuttling	10330 (*n*)<br>150 (*a*)	mM/s
M_{cytox}	Constant for shuttling from cytosol	4.9×10^{-8} (*n*)<br>2.5×10^{-4} (*a*)	-
M_{mitox}	Constant for shuttling from mitochondria	3.93×10^{5} (*n*) 1.06×10^{4} (*a*)	-
$k_{\text{CKon}x}$	Forward rate of creatine kinase	0.0433 (*n*)<br>0.00135 (*a*)	$mM^{-1}s^{-1}$
$k_{\text{CKoff}x}$	Backward rate of creatine kinase	2.8×10^{-4} (*n*)<br>10^{-5} (*a*)	$mM^{-1}s^{-1}$
02_c	O_2 concentration in the bath solution	7	mM
PS_{cap}/V_x	O_2 transport constant from the reservoir	1.66 (*n*)<br>0.87 (*a*)	s^{-1}
K_{O2}	O_2 exchange constant	0.0361	mM
$Hb.OP$	O_2 exchange constant	8.6	mM
n_h	O_2 exchange Hill number	2.73	-
v_x	Relative volumes	0.45 (*n*)<br>0.25 (*a*)	-

		0.20 (*e*)	
ξ	Relative mitochondria volume	0.07	-
$J_{ATPasesx}$	ATPase activity of non-Na-K-ATPases	0.1695 (*n*)<br>0.1404 (*a*)	mM/s
J_{pumpa0}	Basal Na-K-ATPase activity	0 (*n*)<br>0. 0687 (*a*)	mM/s

[1]: after compensation for the slight K^+ permeability of CaL channels, see[41].

[2]: *n* is for the postsynaptic neuronal compartment, *a* for the astrocytic one, *e* for the extracellular pericellular volume and *c* for constant reservoir concentrations.

Tables S3 (related to Figures 2 and 3, *SI Appendix* Figures S4 and S5):

Experimental conditions	5-TBS except for (2)			STDP (50 pairings @ 0.5 Hz) except for (1, 4 and 5)		
	EPSC amplitude % of baseline (n)	t-test, *p* value	2-way ANOVA, *p* value	EPSC amplitude % of baseline (n)	t-test, p value	2-way ANOVA, *p* value
Control, 25mM glucose	169.7 ± 25.7 (8)	0.0299	<0.0001	-	-	-
i-oxamate, 25mM glucose	202.8 ± 20.9 (6)	0.0044	<0.0001	-	-	-
i-Mannoheptulose	134.6 ± 16.8 (7)	0.0230	0.0013	176.7 ± 24.7 (7)	0.0210	<0.0001
Control(1)	-	-	-	151.1±16.8 (8)	0.0191	<0.0001
i-mannoheptulose(1)	-	-	-	176.3±29.9 (6)	0.0436	0.0003
i-Mannoheptulose + i-oxamate(1)	-	-	-	86.6±19.9 (6)	0.5297	0.2856
i-oxamate(1)	-	-	-	151.1±16.8 (8)	0.0191	<0.0001
Control(2)	209.9±33.9 (6)	0.0229	<0.0001	-	-	-
i-oxamate(2)	162.4±15.8 (6)	0.0109	<0.0001	-	-	-
i-oxamate(3)	-	-	-	80.8±9.4 (6)	0.0956	0.0005
i-oxamate+i-NADH(3)	-	-	-	119.4±4.7 (7)	0.0061	0.0004
i-oxamate(4)	-	-	-	228.0±38.8 (6)	0.0216	<0.0001
Control(5)	-	-	-	123.1±8.6 (7)	0.0369	0.0117
i-oxamate(5)	-	-	-	129.0±9.8 (6)	0.0309	<0.0001
Control (6)	-	-	-	97.7±31.5 (6)	0.9439	0.4643
i-oxamate (6)	-	-	-	88.3±21.1 (7)	0.5992	0.0001
Control (mice) (1)	131.9±11.0 (5)	0.0441	0.0002	141.1±14.7 (6)	0.0454	<0.0001
i-oxamate (mice) (1)	70.1±6.8 (5)	0.0121	<0.0001	125.8±3.4 (5)	0.0024	<0.0001

(1) STDP with 100 pairings at 1 Hz, with control *vs* i-oxamate: *p*=0.9836, control *vs* i-oxamate + i-lactate: *p*=0.7095, control *vs* i-oxamate: *p*=0.9837 and i-oxamate *vs* i-oxmate + i-lactate: *p*=0.5901 (t-test)
(2) 1-TBS
(3) STDP with 50 pairings (2bAPs) at 1 Hz
(4) STDP with 25 pairings (2Aps) at 1 Hz
(5) STDP with 25 pairings (2bAPs) at 0.5 Hz
(6) intracellular solution with 2 mM ATP and 5 mM phosphocreatine

Tables S4A-D (related to Figure 4 and SI Appendix Figure S7):

A-

NOR task	Saline-injected rats		Oxamate-injected rats		Saline vs. oxamate
	Time per object (N) vs (F) / Preference index (n)	t-test, *p* value	Time per object / Preference index (n)	t-test, *p* value	t-test, *p* value
All	(N) 66.6±3.1%<br>(F) 33.4±3.1% /<br>66.6±3.1 (9)	0.0007	(N) 67.6±3.2%<br>(F) 32.4±3.2% /<br>67.6±3.2 (10)	0.0003	0.8126
AA-AB	(N) 71.8±2.9%<br>(F) 28.2±2.9% /<br>71.8±2.9 (4)	0.0051	(N) 73.0±4.4%<br>(F) 27.0±4.4% /<br>73.0±4.5 (5)	0.0063	0.8309
BB-BA	(N) 62.4±4.4%<br>(F) 47.6±4.4% /<br>62.4±4.4 (5)	0.0475	(N) 62.3±3.3%<br>(F) 37.7±3.3% /<br>62.3±3.3 (5)	0.0213	0.9792
AA-AB [(1)]	(N) 63.4±4.3%<br>(F) 36.6±4.3% /<br>63.4±4.3 (5)	0.0370	(N) 65.9±2.8%<br>(F) 47.6±2.8% /<br>65.9±2.8 (7)	0.0013	0.7617

[(1)]: rats with chronic recording and stimulation electrodes (e-fEPSPs), with bilateral cannulas.

(N) and (F): novel and familiar, respectively, objects.

B-

OiP task	Saline-injected rats		Oxamate-injected rats		Saline vs. oxamate
	Time per object (N) vs (F) / Preference index (n)	t-test, *p* value	Time per object / Preference index (n)	t-test, *p* value	t-test, *p* value
All	(N) 63.7±2.3%<br>(F) 36.3±2.3% /<br>63.7±2.3 (12)	<0.0001	(N) 46.4±3.0%<br>(F) 53.7±3.0% /<br>46.4±3.0 (9)	0.2500	0.0002
AC	(N) 68.6±3.2%<br>(F) 31.4±3.2% /<br>68.6±3.2 (6)	0.0020	(N) 53.0±4.7%<br>(F) 46.9±4.7% /<br>53.0±4.7 (4)	0.5603	0.0209
BD	(N) 58.9±2.0%<br>(F) 41.1±2.0% /<br>58.9±2.0 (6)	0.0068	(N) 41.0±1.3%<br>(F) 59.0±1.3% /<br>41.0±1.3 (5)	0.0026	<0.0001
AC [(1)]	(N) 61.7±1.2%<br>(F) 38.3±1.2% /<br>61.7±1.2 (9)	<0.0001	(N) 49.1±1.7%<br>(F) 50.9±1.7% /<br>49.1±1.7 (12)	0.6239	<0.0001

[(1)]: rats with chronic recording and stimulation electrodes (e-fEPSPs), with bilateral cannulas.

(N) and (F): novel and familiar, respectively, objects.

C- Evoked-fEPSP plasticity at 2 and 24 hours after familiarization phase in saline- and oxamate-injected rats in NOR and OiP tasks.

e-fEPSP monitoring	Saline-injected rats		Oxamate-injected rats		Saline *vs.* oxamate
	t post-familiarization: +2 h (n) + 24 h (n)	t-test, *p* value	*t* post-familiarization: +2 h (n) + 24 h (n)	t-test, *p* value	t-test, *p* value
NOR task	108.2±6.4 (5)	0.2686	128.0±10.7 (7)	0.0395	0.1839
	96.1±6.7 (5)	0.5904	112.1±15.3 (7)	0.4570	0.4210
OiP task	114.9±5.6 (7)	0.0377	93.7±6.3 (9)	0.3447	0.0287
	71.8±15.7 (7)	0.0778	87.3±6.6 (12)	0.0471	0.2380

D- Comparison of average vectors in saline- and oxamate-injected rats (MANCOVA)

Average vectors in saline- and oxamate-injected rats (MANCOVA)	Univariate test on the e-fEPSP plasticity axis		Univariate test on the behavior axis		Multivariate Test	
	F	*p* value	F	*p* value	F	*p* value
NOR task						
+2h	2.038	0.184	0.260	0.621	0.980	0.412
+24h	0.704	0.421	0.260	0.621	0.353	0.712
OiP task						
+2h	5.94	0.029	16.43	0.01	14,7	<0.001
+24h	1.50	0.238	28.82	< 0.001	13.9	<0.001

SI References

1. Feldman, D. E. The spike-timing dependence of plasticity. Neuron 75, 556–571 (2012).
2. Xu, H. et al. Dopamine-endocannabinoid interactions mediate spike-timing-dependent potentiation in the striatum. Nat Commun 9, 1, 4118 (2018).
3. Graupner, M. & Brunel, N. Calcium-based plasticity model explains sensitivity of synaptic changes to spike pattern, rate, and dendritic location. Proc Natl Acad Sci U S A 109, 10, 3991-6 (2012).
4. R. Jolivet, J. S. Coggan, I. Allaman, P. J. Magistretti, Multi-timescale modeling of activity-dependent metabolic coupling in the neuron-glia-vasculature ensemble. PLoS Comput Biol 11, 2, e1004036 (2015).
5. Roberts, P. AMPA Glutamate Receptor (AMPA Receptor), Conductance Models. In Jaeger D., Jung R. (eds) *Ency Comput Neurosci* 2014 Springer, New York, NY (2014).
6. Roberts, P. N-Methyl-D-Aspartate (NMDA) Receptors, Conductance Models. In: Jaeger D., Jung R. (eds) *Ency Comput Neurosci*, 2014 Springer, New York, NY (2014).
7. Solinas, S., Masoli, S. & Subramaniyam, S. High-Voltage-Activated Calcium Channels. In Jaeger D., Jung R. (eds) *Ency Comput Neurosci* 2014 Springer, New York, NY. (2014).
8. Migliore, M., Ferrante, M. & Ascoli, G. A. Signal propagation in oblique dendrites of CA1 pyramidal cells. *J Neurophysiol* 94, 6, 4145-55 (2005).
9. Watanabe, S., Hoffman, D. A., Migliore, M. & Johnston, D. Dendritic K+ channels contribute to spike-timing dependent long-term potentiation in hippocampal pyramidal neurons. Proc Natl Acad Sci U S A 99, 12, 8366-71 (2002).
10. Sabatini, B. L., Oertner, T. G. & Svoboda, K. The life cycle of Ca(2+) ions in dendritic spines. *Neuron* 33, 3, 439-52 (2002).
11. Carter, A. G. & Sabatini, B. L. State-dependent calcium signaling in dendritic spines of striatal medium spiny neurons. *Neuron* 44, 3, 483-93 (2004).
12. K. A. Kasischke, H. D. Vishwasrao, P. J. Fisher, W. R. Zipfel, W. W. Webb, Neural activity triggers neuronal oxidative metabolism followed by astrocytic glycolysis. *Science* 305, 5680, 99-103 (2004).
